# Multidimensional Dynamic Control of Supramolecular Phthalocyanine Gear: A Self-Assembly System Responding to Solvent, Temperature, and Hydrostatic Pressure

**DOI:** 10.1021/acsomega.4c03584

**Published:** 2024-07-31

**Authors:** Tomokazu Kinoshita, Daisuke Sakamaki, Gaku Fukuhara

**Affiliations:** † Department of Chemistry, Tokyo Institute of Technology, 2-12-1 Ookayama, Meguro-ku, Tokyo 152-8551, Japan; ‡ Department of Chemistry, Graduate School of Science, Osaka Metropolitan University, Sumiyoshi-ku, Osaka 558-8585, Japan

## Abstract

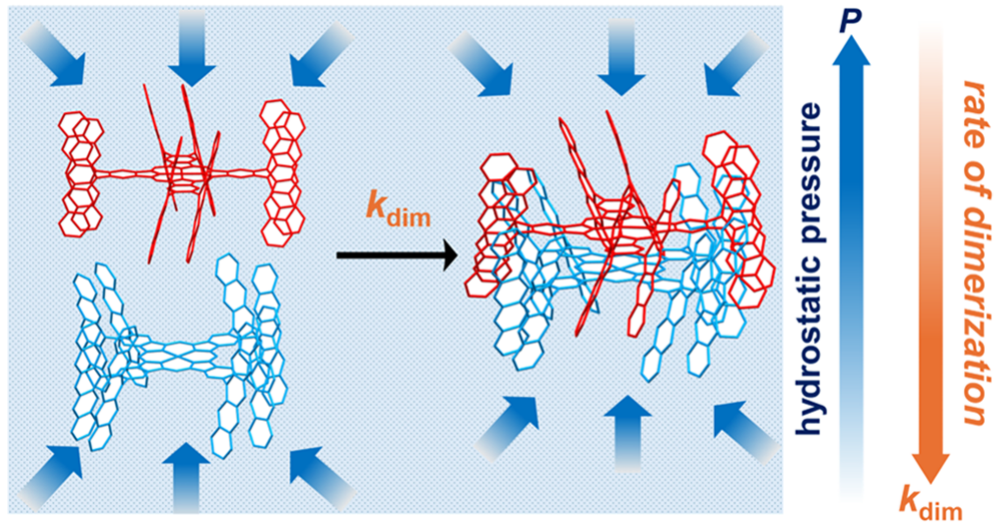

Smart supramolecular materials that respond toward various external stimuli hold great promise for various applications in molecular memories, logic gates, and drug delivery systems. In this study, the active control over the self-assembly of phathalocyanine gear was achieved by combining temperature and hydrostatic pressure stimuli with a dynamic solvent. Eventually, we found that the supramolecular gear can behave as a logic gate; “engaged” (+1) or “not” (0) state is switchable by solvent, temperature, and hydrostatic pressure. This paper describes not only new aspects for the rational design of smart stimuli-responsive supramolecular materials but also the significance of multidimensional dynamic control.

## 1 Introduction

Supramolecular materials that respond to a large variety of external stimuli such as solvent, temperature, photons, pH, and mechanical forces (including pressure, stress, strain, and tension) have attracted considerable attention in the field of multidisciplinary chemistry.^1−8^ Such smart materials have potential applications in molecular memories, logic gates, and drug delivery systems, which require stimuli-responsive structural/optical/functional changes.^9−12^ The current mainstream for creating supramolecular materials undoubtedly represents a “bottom-up” approach rather than the “top-down” one with the growing field of supramolecular and supramolecular polymer chemistry.^13−17^ In general, “nature” assembles a functional monomer in a smart manner to produce complex but highly ordered structures, such as proteins and enzymes.^18,19^ Namely, it provides a valuable hint that self-assembly (particularly self-dimerization) is the most effective and fundamental bottom-up approach. Hence, incorporating stimuli-responsiveness into the self-dimerization process can lead to the creation of smart supramolecular materials with good responsiveness. This stimulates the exploration of a novel approach toward the discovery of stimuli-responsive supramolecular materials.

In recent years, hydrostatic pressure or solution-state isotropic pressure used as an external stimulus, has become the focus of many researchers^20^ because its effect on mechanochemical materials^21^ and mechanobiological living systems^22^ have not been fully explored yet; hence, we excluded the high-pressure solid chemistry studies conducted using a diamond anvil cell (∼GPa),^23^ which is beyond the targeted pressure range during hydrostatic pressurization (∼MPa). Historically, hydrostatic pressure effects in solutions have been investigated since the 1960s.^24−33^ Nevertheless, few studies on the influence of hydrostatic pressure on the self-assembly process have been conducted up to date. Recently, we have reported the solution-state supramolecular polymerization of curved-π sumanene buckybowls and attempted to implement the hydrostatic pressure control of supramolecular polymerization.^34^ Unfortunately, the effect of hydrostatic pressurization was negligible, as indicated by the small Δ*V*° value of 2.7 cm^3^ mol^–1^ due to dense stacking. Thus, a self-assembly system stimulated by hydrostatic pressure should be developed.

**Figure 1:**
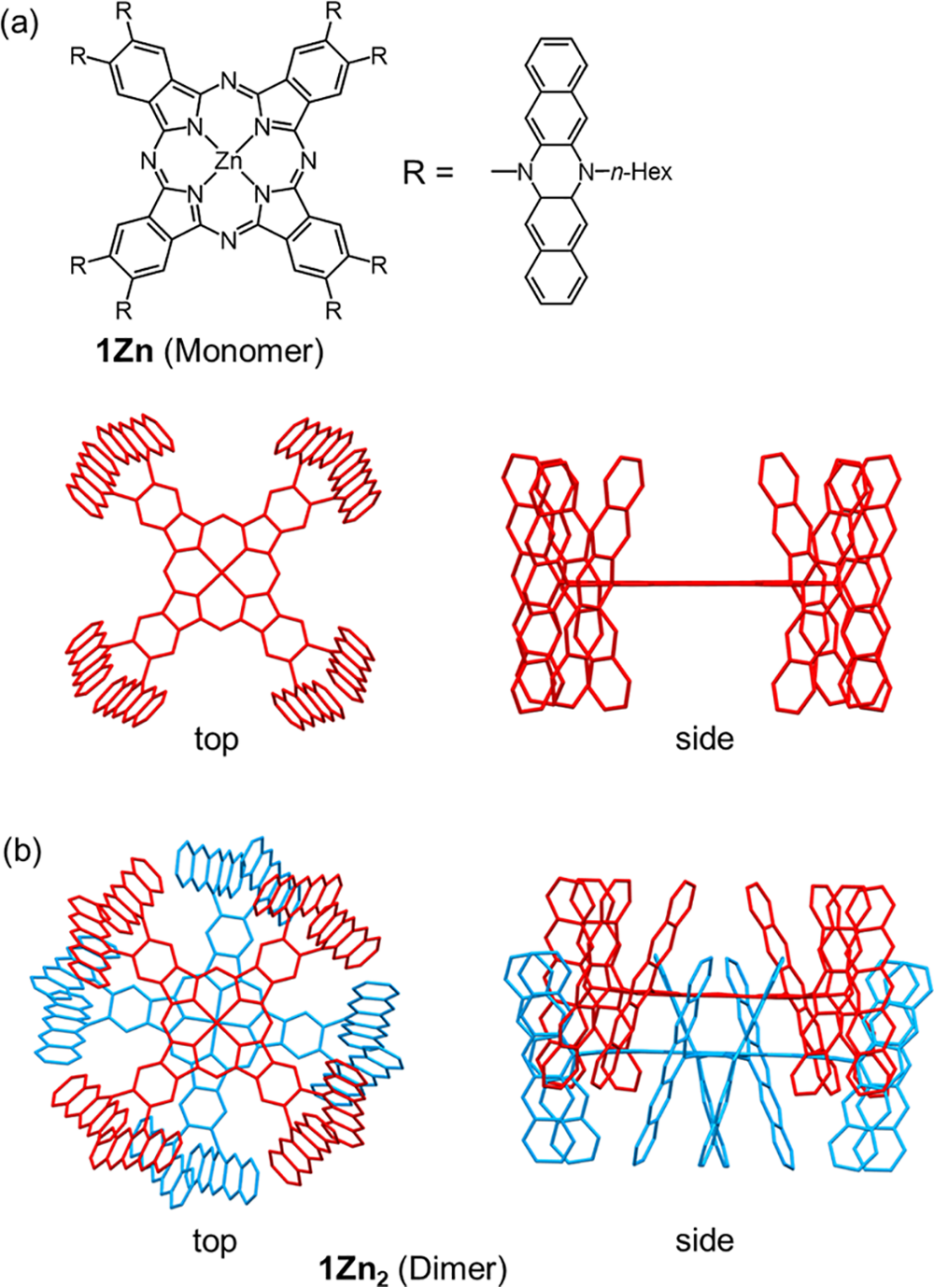
Structures of the phthalocyanine derivative: (a) monomer (**1Zn**) and (b) dimer (**1Zn**_**2**_). *n*-Hexyl groups were omitted for clarity. 3D models were obtained by the DFT-optimization for the model compounds without *n*-hexyl groups.

In this study, we focused on the supramolecular self-dimerization of Zn-coordinated phthalocyanine (Pc) containing four pairs of 6,13-dihydro-6,13-diazapentacene (DHDAP) pillars with *n*-hexyl side chains on the periphery (**1Zn**),^35^ as shown in Figure 1. **1Zn** forms an *H*-type dimer **1Zn**_**2**_ via π–π interactions between two cofacial Pc rings and interdigitated DHDAP pillars in specific solvents at ambient pressure (0.1 MPa), as if a “gear” is perfectly engaged. Figure 1 shows the optimized structures of the model compounds of **1Zn** and **1Zn**_**2**_ without *n*-hexyl side chains (**1Zn’** and **1Zn’**_**2**_) at the M06-2X/6-31G(d) (for H, C, N) and LANL2DZ (for Zn) level of theory (Cartesian geometries are shown in SI). The stabilization energy upon the dimerization was calculated to be approximately 400 kJ mol^–1^, which is comparable to that of the covalent bond formation. Two **1Zn’** molecules are closely engaged by filling each other’s voids with DHDAP pillars, and nearly three of the five six-membered rings in each DHDAP unit are inserted into the voids of the counterpart monomer. The close contact of the two Zn atoms (3.315 Å) suggests the existence of a strong attractive interaction between the two **1Zn’** molecules. In addition to the π–π stacking between two Pc planes, some intermonomer short contacts (<3.4 Å) between the DHDAP units were found, indicating the existence of intermonomer π–π interactions among the pillars. In our previous study, we successfully isolated **1Zn** and **1Zn**_**2**_, as well as their Cu-analogues **1Cu** and **1Cu**_**2**_, and thoroughly investigated their structures.^35^ By using mass spectrometry and spectroscopic measurements, including various 1D and 2D NMR techniques (^1^H–^1^H COSY, ^13^C DEPT, ^13^C/^1^H HMBC, ^13^C/^1^H HMQC, and 1D DPFGSE-NOE) for **1Zn** and **1Zn**_**2**_ and ESR measurements for **1Cu** and **1Cu**_**2**_, we confirmed that the dimers possess *D*_4_ symmetric structures where the DHDAP pillars are interdigitated to the voids of the counterpart monomer. Interestingly, **1Zn** underwent only dimerization and did not form higher oligomers.^35^ The absence of trimers and higher oligomers was explained by the optimized structure of the dimer, in which no sufficient space was left for the interdigitation of the pillars of the third monomer (Figure S16).

The most important characteristics of **1Zn** after dimerization is a strong solvent dependency.^35^ In tetrahydrofuran (THF), both **1Zn** and **1Zn**_**2**_ existed as stable species that were separated from each other, and no interconversion was observed even at 333 K. The results indicated that not only the dissociation of **1Zn**_**2**_ but also the dimerization of **1Zn** requires significant activation energies owing to the steric demand upon the interdigitation of the pillars. **1Zn** exhibited an intense absorption band at 675 nm corresponding to the Q-band of Pc in THF, which hypsochromically shifted to 652 nm upon the dimerization to **1Zn**_**2**_ owing to the *H*-type configuration of the two Pc units. In dichloromethane, toluene, and *o*-dichlorobenzene, **1Zn**_**2**_ was in a metastable state that gradually dissociated to produce monomer **1Zn**. In contrast, in ethyl acetate (EA), monomer **1Zn** transformed to a metastable state and then dynamically dimerized (**1Zn**_**2**_). These results indicate that the solvation core in **1Zn** plays a critical role in the dimerization process, revealing a highly dynamic nature during self-assembly. Such a dynamic dimerization system of **1Zn** becomes the most viable candidate for the purpose of the present work. Herein, we report a novel stimuli-responsive supramolecular “gear”, **1Zn,** that can be dynamically controlled by varying both the hydrostatic pressure and temperature in the dynamic EA solution. It was found that all cooperative factors, including the multidimensional dynamic control by the solvent, temperature, and hydrostatic pressure, are essential for the self-assembly process. For example, among the representative self-dimerization systems about the oligomeric strands,^36^ expanded helicenes,^37^ and merocyanine dyes,^38^ temperature and/or solvent appear to be effective control factors.

## 2 Experimental Section

### Instruments

Ultraviolet/visible/near-infrared (UV/vis/NIR) absorption spectra were recorded using a JASCO V-770 spectrometer.

### Materials

Spectrophotometric grade THF, toluene, dichloromethane, and ethyl acetate were used as received without further purification. **1Zn** and **1Zn**_**2**_ were synthesized according to the literature.^35^

### Hydrostatic Pressure Spectroscopy

UV/vis/NIR absorption spectra were recorded under the hydrostatic pressure using a custom-built high-pressure apparatus.^20^ Because the utilized method was previously described in detail, we outline it only briefly. A quartz inner cell was filled with a sample solution and then placed into an outer cell with fitted sapphire windows. A tightly closed outer cell was hydrostatically pressurized using water and then placed inside the spectrometer to obtain the hydrostatic pressure spectra presented in this paper.

## 3 Results and Discussion

### Dynamic Self-Assembling of the Gear Induced by Temperature Stimulus

**Figure 2:**
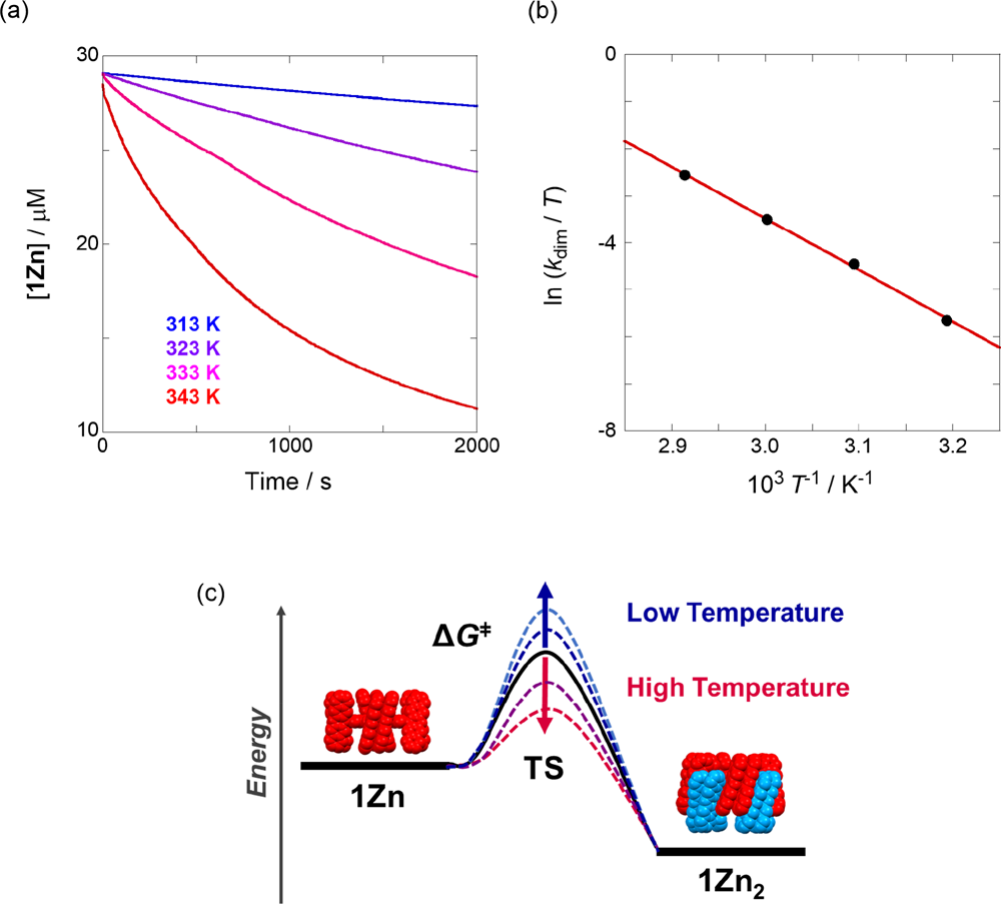
(a) Time-dependent concentration changes of **1Zn** in EA observed at 0.1 MPa. Temperature applied: 313, 323, 333, and 343 K (from blue to red). (b) Eyring plot of the dimerization process of **1Zn** (29 μM) in EA conducted at 0.1 MPa *(r* = 0.999). (c) Energy diagram of the dimerization in EA conducted at 0.1 MPa.

First, before studying the effect of hydrostatic pressurization, we investigated the influence of the temperature stimulus on the dimerization process (particularly in EA) through a metastable state. The temperature-dependent UV/vis/NIR spectra of **1Zn** recorded at 313–343 K exhibit distinct shifts to **1Zn**_**2**_, for which no changes were observed under the same conditions. This indicates the irreversibility of the conversion from **1Zn** to **1Zn**_**2**_ with a significant role of temperature. Thus, the dimerization rate constant *k*_dim_ can be expressed as follows:
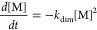
1
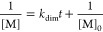
2where [M]_0_ and [M] represent the monomer concentrations at the initial time and arbitrary time (*t*), respectively. Upon applying temperature in the range of 313–343 K to an EA solution of **1Zn** (29 μM), the time-course absorbance monitored at the Q-band (675 nm) decreased (Figure S1 in SI). Combining Figure S1 and eq 2 enabled fitting the data presented in Figure S3, which resulted in straight lines (correlation coefficients: *r* = 0.999). The slopes in Figure S3 represent the *k*_dim_ values obtained at four temperatures (Table S1). Moreover, a similar temperature dependence was observed for the time-course **1Zn** concentration changes (Figure 2a). To quantitatively evaluate the enthalpic and entropic contributions to the self-assembly process, the *k*_dim_ data obtained at the four temperatures were subjected to the Eyring analysis procedure (Figure 2b). For this purpose, the natural logarithm of *k*_dim_ was plotted as a function of the reciprocal temperature. According to Figure 2b, each set of data points falls on a single straight line (*r* = 0.999), indicating that the self-dimerization mechanism did not change in the tested temperature range. The activation enthalpy Δ*H*^‡^ of 91.5 kJ mol^–1^ and entropy Δ*S*^‡^ of 47.9 J K^–1^ mol^–1^ were estimated from the slope and intercept of the straight line, and the activation Gibbs free energy Δ*G*^‡^ of 77.2 kJ mol^–1^ (298 K) was calculated using the two kinetic parameters. The positive Δ*G*^‡^ value indicates that dimerization does not proceed spontaneously at room temperature; however, elevating the temperature promotes the dimerization process by exceeding the activation energy in the transition state (TS), which is a critical factor for engaging each “gear” (Figure 2c). In fact, the Δ*G*^‡^ value observed here necessitates a temperature condition at least over 313 K for the spontaneous dimerization, as shown in Figure 2a. Among the main Δ*G*^‡^ components, the positive Δ*S*^‡^ value drives the dimerization process. This phenomenon can be reasonably explained considering that the broad-range solvent EA core around monomer **1Zn** is highly likely to be blown off along with the gear formation in the TS (desolvation). The desolvation process leads to the endothermic reaction (positive Δ*H*^‡^) in the TS although various gear pieces fully engage with each other. This result indicates that solvation (for **1Zn**) or desolvation (for **1Zn**_**2**_) plays a decisive role in the self-assembly process, confirming its dynamic nature.

### Hydrostatic Pressure Stimulus Controls the Gear Formation

Next, we investigated the gear responses to the hydrostatic pressure stimulus. In this experiment, UV/vis/NIR absorption spectra were recorded at high pressures up to 320 MPa and room temperature (298 K) using a previously reported optical system (see Experimental Section). Measurements were performed from the NIR (1300 nm) to UV (300 nm) region in THF, toluene, dichloromethane, and EA. Gradual pressure-induced bathochromic shifts and hyperchromic effects were observed for both **1Zn** and **1Zn**_**2**_ (Figures S4 and S7 in SI, respectively). The former behavior originates from the pressure-induced solvent polarizability (density) changes that cause the stabilization of the π* orbital,^24,25^ and the latter phenomenon occurs because of the increasing effective concentration during solution compression. Considering that the hydrostatic pressure effect on the bathochromic shift is generally observed at approximately 1 cm^–1^ MPa^–1^ for common π-conjugated organic molecules (such as anthracene, pyrene, and perylene),^20,39−41^ the resulting shifts of 0 ∼ −0.39 cm^–1^ MPa^–1^ for **1Zn** and −0.11 ∼ −0.25 cm^–1^ MPa^–1^ for **1Zn**_**2**_ were relatively small (Figures S6 and S9 in SI, respectively). This remarkable pressure effect was likely responsible for the characteristic Q-band obtained for the Pc chromophore, which revealed that the hydrostatic pressure responses in the Q-band were considerably different from those obtained for the regular π–π* transition. In addition, the normalized UV/vis/NIR spectra of **1Zn**_**2**_ and **1Zn** in all solutions obtained at the hydrostatic pressure are almost superimposable (Figures S5 and S8 in SI, respectively), indicating that hydrostatic pressurization did not cause dissociation/association, particularly in specific solvation EA. These hydrostatic pressurization experiments conducted at 298 K ultimately enable dynamic control by combining the temperature and hydrostatic pressure stimuli (multidimensional control).

**Figure 3:**
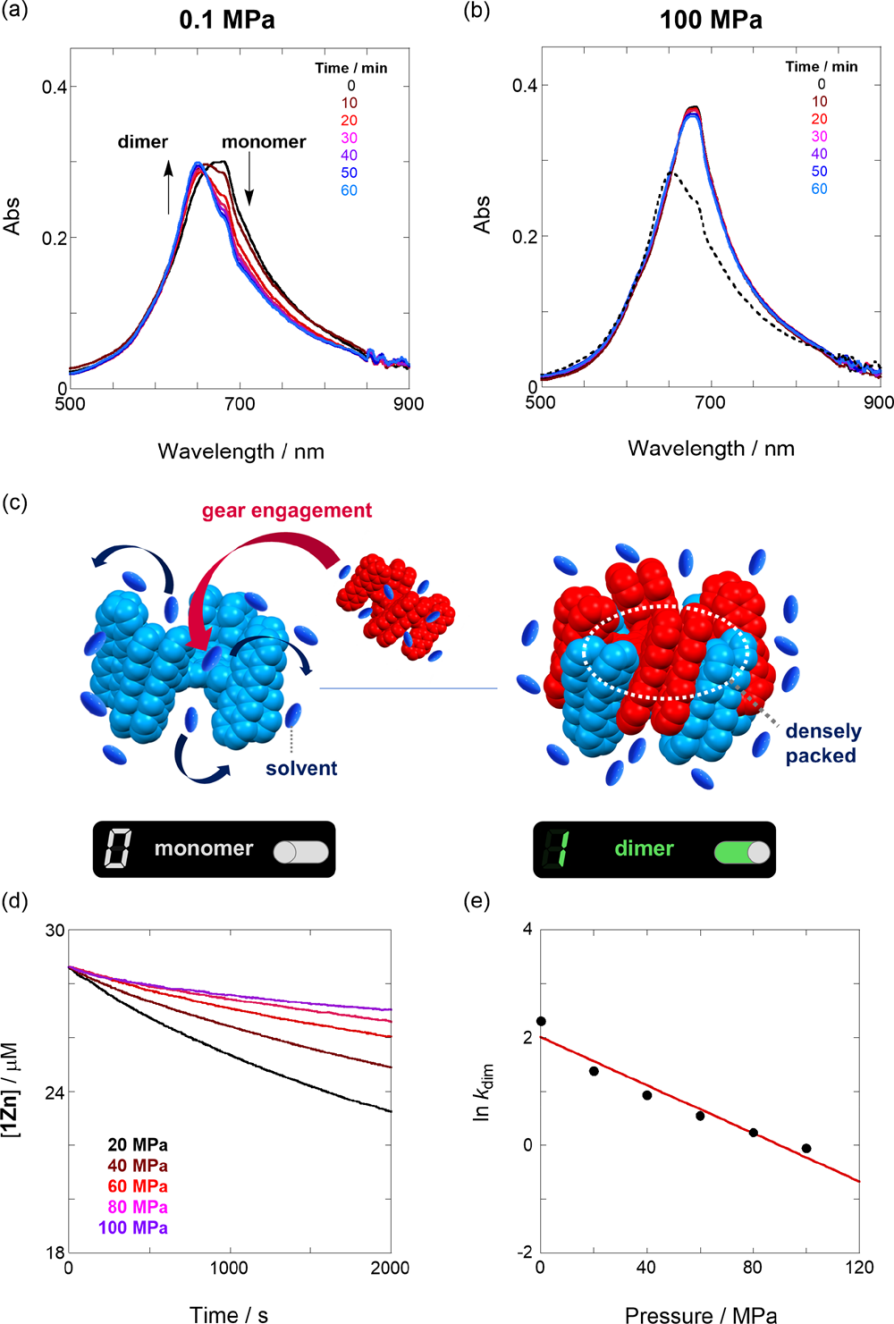
Time-dependent UV/vis/NIR absorption spectra of **1Zn** (32 μM) obtained in EA at a temperature of 333 K and pressures of (a) 0.1 MPa (in a 2 mm cell) and (b) 100 MPa (in a high-pressure cell). The colored lines represent the spectrum after standing at 0, 10, 20, 30, 40, 50, and 60 min (from black to light blue). The black dotted line in (b) represents the depressurized spectrum recorded after applying a pressure of 100 MPa for 60 min and then 0.1 MPa for 20 min. (c) Dimerization mechanism of **1Zn**. (d) Pressure-dependent concentration changes of **1Zn** observed in EA at a temperature of 333 K and various pressures of 20, 40, 60, 80, and 100 MPa (from black to purple). (e) Pressure dependence of *k*_dim_ obtained for EA at 333 K (*r* = 0.972).

Thus, we investigated the hydrostatic pressure effects on the dimerization process in the dynamic EA solvent at an appropriate temperature. According to Figure 3a, spectral measurements were conducted every 10 min at 333 K under 0.1 MPa. As a result, the absorbance in the monomeric band (675 nm) continuously decreased, and a dimeric band (649 nm) appeared; the dimerization process spontaneously occurred under these conditions (*vide supra*). Meanwhile, when a similar experiment was performed at a hydrostatic pressure of 100 MPa, the monomer band at 0 min remained nearly intact during pressurization (Figure 3b, colored lines). This means that the pressurized monomer does not dimerize. After applying a pressure of 100 MPa for 60 min, the pressurized system was depressurized to the original atmospheric pressure, and the spectrum obtained in 20 min contained the dimer band (Figure 3b, black dotted line). This indicates that the supramolecular gear is either “engaged” (+1 state) or “not engaged” (0 state) depending on the hydrostatic pressure (Figure 3c).

To finally elucidate the governing mechanism for the stimuli-responsive supramolecular gear, we investigated the high-temperature dimerization kinetics (*k*_dim_) under the hydrostatic pressure in the dynamically solvated EA. The time-course absorbance curves recorded in 20 MPa increments from 20 to 100 MPa (Figure S10) produced the pressure-dependent *k*_dim_ constant (the fitting results are presented in Figure S15, and the obtained data are summarized in Table S2). In addition, the time-course **1Zn** concentration changes (Figure 3d) clearly depend on the applied pressure.

The correlation between the activation volume (Δ*V*^‡^) and kinetic constant can be expressed as follows:
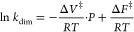
3

According to eq 3, the natural logarithm of *k*_dim_ plotted against *P* (Figure 3e) results in a straight line (*r* = 0.972), indicating that in this pressure range, the hydrostatic pressure effect does not change the dimerization TS. The Δ*V*^‡^ value estimated from the slope is equal to +62.0 cm^3^ mol^–1^, which is larger than those reported previously for pressure-responsive chemosensor systems (<10 cm^3^ mol^–1^) owing to the desolvation/solvation processes Δ*V*^20,42^ and above-mentioned sumanene-based supramolecular polymerization. Hence, such a large change in Δ*V*^‡^ can be easily explained by not the regular solvation process but the highly dense or “expanded” (positive Δ*V*) gear engaging during dimerization (Figure 3c). This gear expansion scenario in the TS was further supported by the above-mentioned positive Δ*S*^‡^ value; the dynamic solvation core around the monomer is excluded (positive Δ*S*^‡^) to open up an effective space that enables the perfect engagement of each gear (positive Δ*V*^‡^). Incidentally, the limitation of this multidimensional dynamic control was based on the boiling point of EA (77 °C) and the upper limitation of our high-pressure apparatus (∼400 MPa).

## 4 Conclusions

In conclusion, a smart dynamic stimuli-responsive supramolecular gear has been discovered for the first time. The gear worked the temperature- and volume-correlated Δ*G*^‡^ as 77.2 kJ mol^–1^ and Δ*V*^‡^ as +62.0 cm^3^ mol^–1^, both of which were mutually compensated in dynamic EA solvent. Such dynamic responses were achieved by combining the temperature and hydrostatic pressure stimuli in the dynamic solvation shell, which can be considered *multidimensional dynamic control*. Our findings not only provide new guidelines for exploring smart materials that respond to various stimuli but also proposes a dynamic control concept using applicable external stimuli. For further expanding the multidimensional dynamic control concept, systems that are controlled by temperature, solvent, pressure, and other stimuli are currently in progress.

## Supporting Information Available

Calculation method, temperature-dependent kinetics, hydrostatic pressure spectroscopy, pressure-dependent kinetics, and computational simulations (PDF)
