# Supplementary material for: Multidimensional Dynamic Control of Supramolecular Phthalocyanine Gear: A Self-Assembly System Responding to Solvent, Temperature, and Hydrostatic Pressure
Source: ACS Omega. 2024 Jul 31;9(32):34719–24. doi: 10.1021/acsomega.4c03584 (PMC11325503; doi:10.1021/acsomega.4c03584)
Supplement: Supplementary file 1 — ao4c03584_si_001.pdf [file ao4c03584_si_001.pdf]

Supporting Information
for

Multidimensional Dynamic Control of Supramolecular Phthalocyanine Gear: A Self-assembly System Responding to Solvent, Temperature, and Hydrostatic Pressure

***Tomokazu Kinoshita,*[a] *Daisuke Sakamaki**[*,b] *and Gaku Fukuhara**[*,a]

[a]Department of Chemistry, Tokyo Institute of Technology, 2-12-1 Ookayama, Meguro-ku, Tokyo 152-8551, Japan

[b]Department of Chemistry, Graduate School of Science, Osaka Metropolitan University, Sumiyoshi-ku, Osaka 558-8585, Japan

*E-mail: sakamaki@omu.ac.jp (D.S); gaku@chem.titech.ac.jp (G.F.)

Calculation method for the kinetic constant

Assuming that the dimerization of two monomers is an irreversible process, the dimerization rate constant can be expressed as k_{dim}. The monomer concentrations at times $t = 0$ and t are equal to $[\mathrm{M}]$ and $[\mathrm{M}]_0$, respectively.

$$\frac{d[\mathrm{M}]}{dt} = -\,k_{dim}[\mathrm{M}]^2 \quad \text{(S1)}$$

$$\frac{1}{[\mathrm{M}]} = k_{dim}t + \frac{1}{[\mathrm{M}]_0} \quad \text{(S2)}$$

At $t = 0$, ε_{M} and l represent the molar extinction coefficient of the monomer and cell length, respectively.

$$Abs_0 = \varepsilon_{\mathrm{M}}[\mathrm{M}]_0 l \quad \text{(S3)}$$

At $t = t$, ε_D and [D] represent the molar extinction coefficient of the dimer and dimer concentration, respectively.

$$Abs_t = \varepsilon_{\mathrm{M}}[\mathrm{M}]l + \varepsilon_{\mathrm{D}}[\mathrm{D}]l \ \text{(S4)}$$

$$[\mathrm{D}] = \frac{1}{2}\left([\mathrm{M}]_0 - [\mathrm{M}]\right) \quad \text{(S5)}$$

Using Eqs. (S3)–(S5) and $\varepsilon_{\mathrm{D}} \,/\, 2\varepsilon_{\mathrm{M}} = C$,

$$\frac{1 - C}{1 - \frac{Abs_t}{Abs_0}} \cdot \frac{1}{[\mathrm{M}]_0} = k_{dim}t + \frac{1}{[\mathrm{M}]_0} \quad \text{(S6)}$$

ε_{M} and ε_{D} were estimated by extrapolating the calibration curves obtained in the temperature range of 283–313 K (Figure S2).

Using Eq. (S6), the fitting results presented in Figure S3 were obtained to calculate k_{dim} at 0.1 MPa (Table S1).

Temperature-dependent kinetics

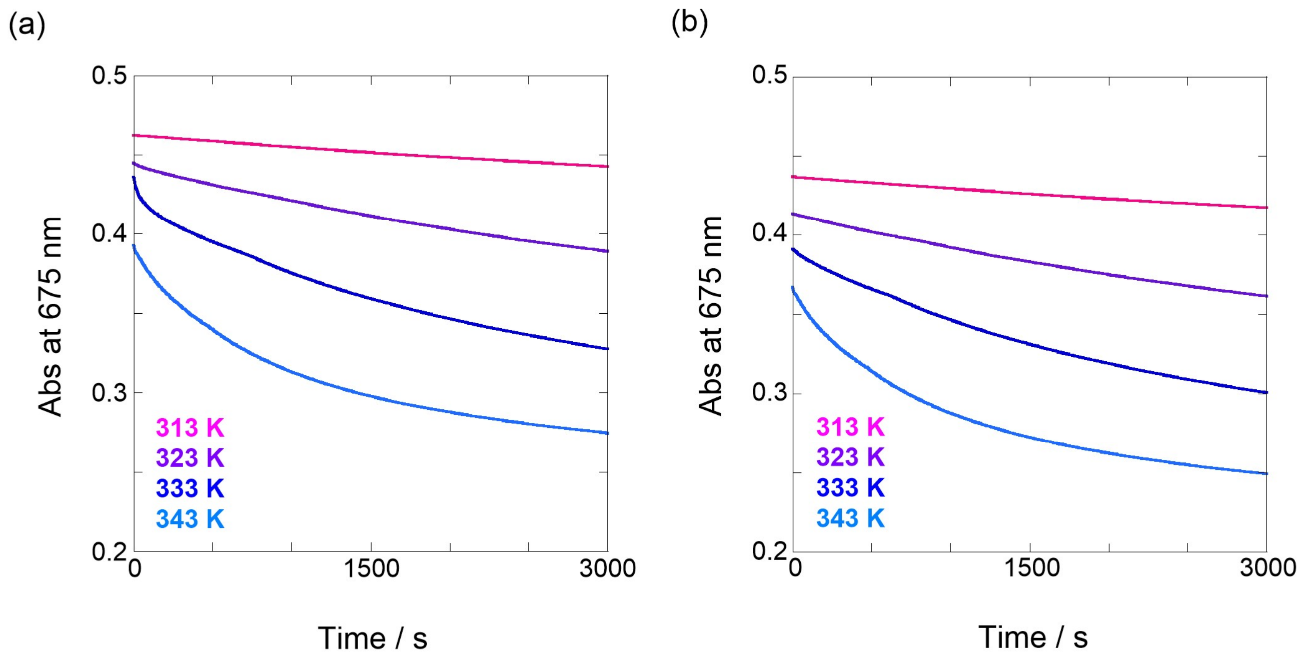

Figure S1. (a) Temperature-dependent UV/vis/NIR spectroscopic time-course curves of **1Zn** (29 μM) obtained during dimerization in ethyl acetate at a wavelength of 675 nm and applied pressure of 0.1 MPa. Temperature applied: 313, 323, 333, and 343 K (from pink to light blue). (b) Corrected spectra displayed in (a).

$Abs_{313K\_cal(t=0)}$ (estimated from Figure S2) and $Abs_{313K\_app(t=0)}$ (shown in Figure S1a) represent the monomer absorbance values estimated by extrapolating the calibration curve and actual values, respectively.

$$\Delta Abs_{\ T} = Abs_{313K\_app(t=0)} - Abs_{313K\_cal(t=0)}$$

By subtracting $\Delta Abs_{\ T}$ from the actual value,

$$Abs'_{T\_app(t)} = Abs_{T\_app(t)} - \Delta Abs_{\ T}$$

we determined the starting time ($t = 0$), at which $Abs'_{T\_app(t)} = Abs_{313K\_cal(t=0)}$ to produce the corrected data presented in Figure S1b.

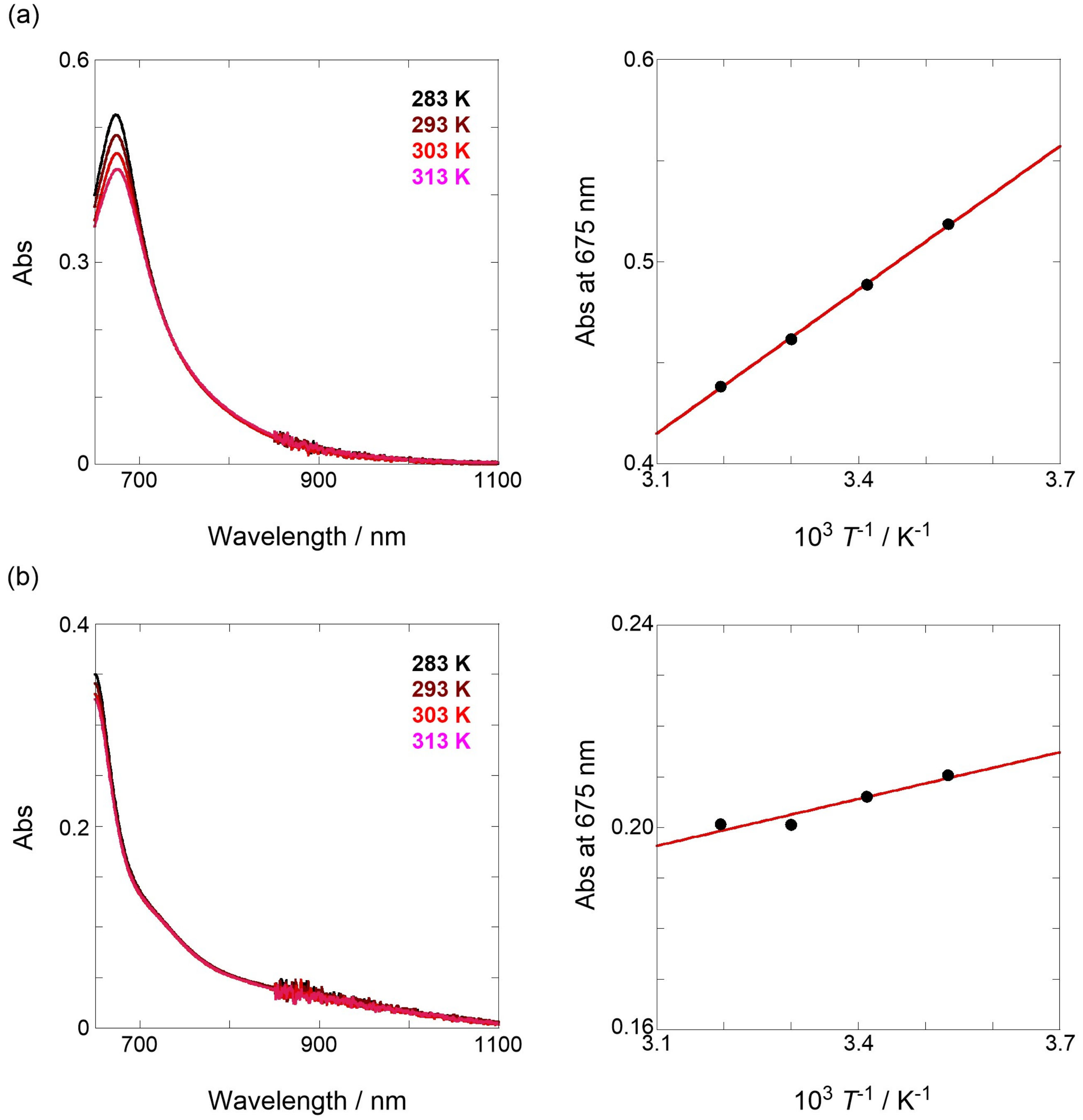

Figure S2. Temperature-dependent UV/vis/NIR spectra (*left*) and absorbance plots at 675 nm (*right*) obtained for (a) **1Zn** (29 µM, correlation coefficient r = 0.999) and (b) **1Zn₂** (14 µM, r = 0.951) in ethyl acetate at 0.1 MPa inside the 2-mm cell. Temperature applied: 283, 293, 303, and 313 K (from black to pink).

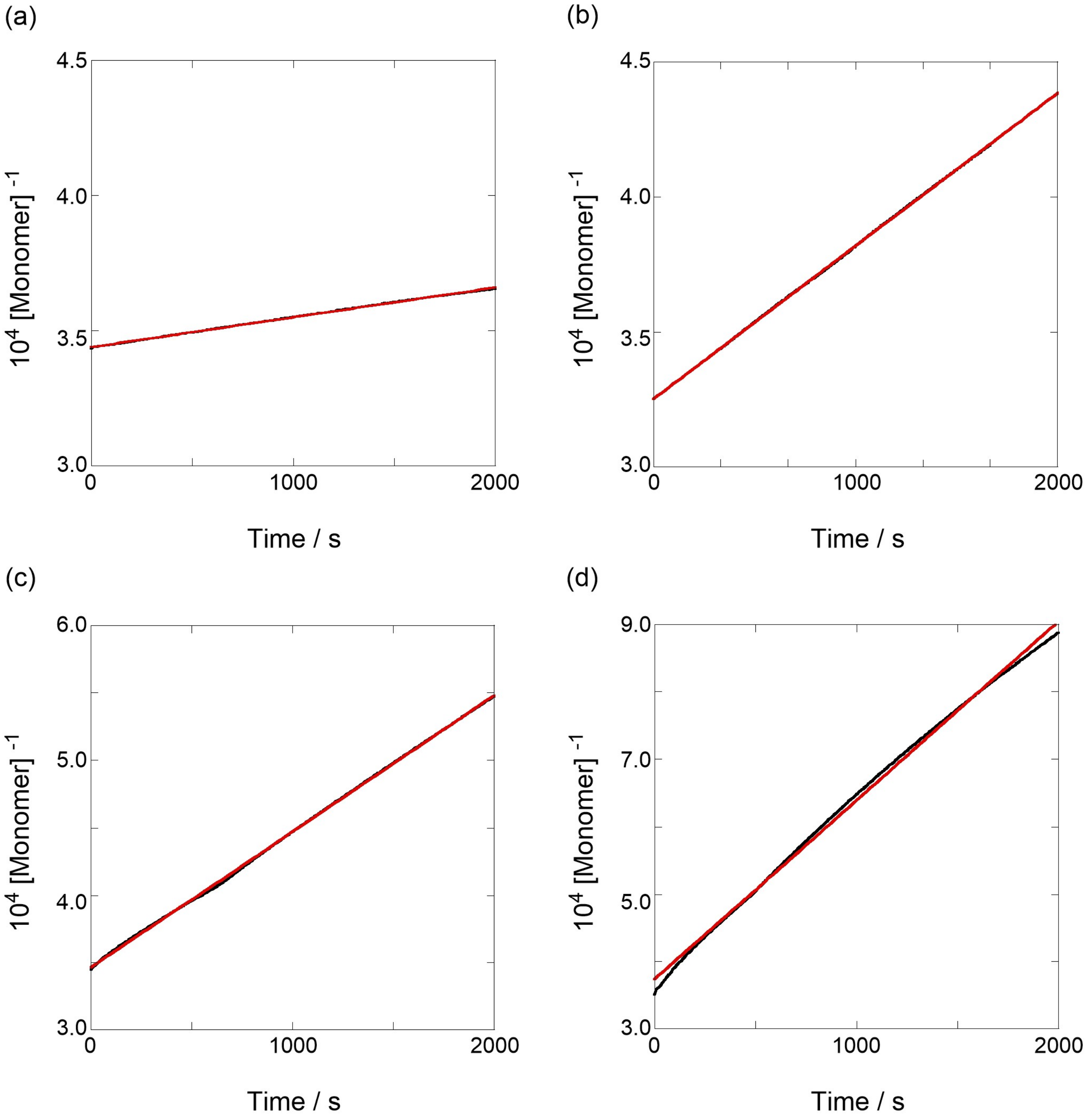

Figure S3. Time-dependent 1/[Monomer] plots obtained in ethyl acetate at ambient pressure and temperatures of (a) 313 K (r = 0.999), (b) 323 K (r = 0.999), (c) 333 K (r = 0.999), and (d) 343 K (r = 0.999).

Table S1. Dimerization rates of 1Zn in ethyl acetate at 0.1 MPa

T / K	313	323	333	343
k_{dim} / M^{-1} s^{-1}	1.1	3.8	10.1	26.6

Hydrostatic pressure spectroscopy conducted at 298 K

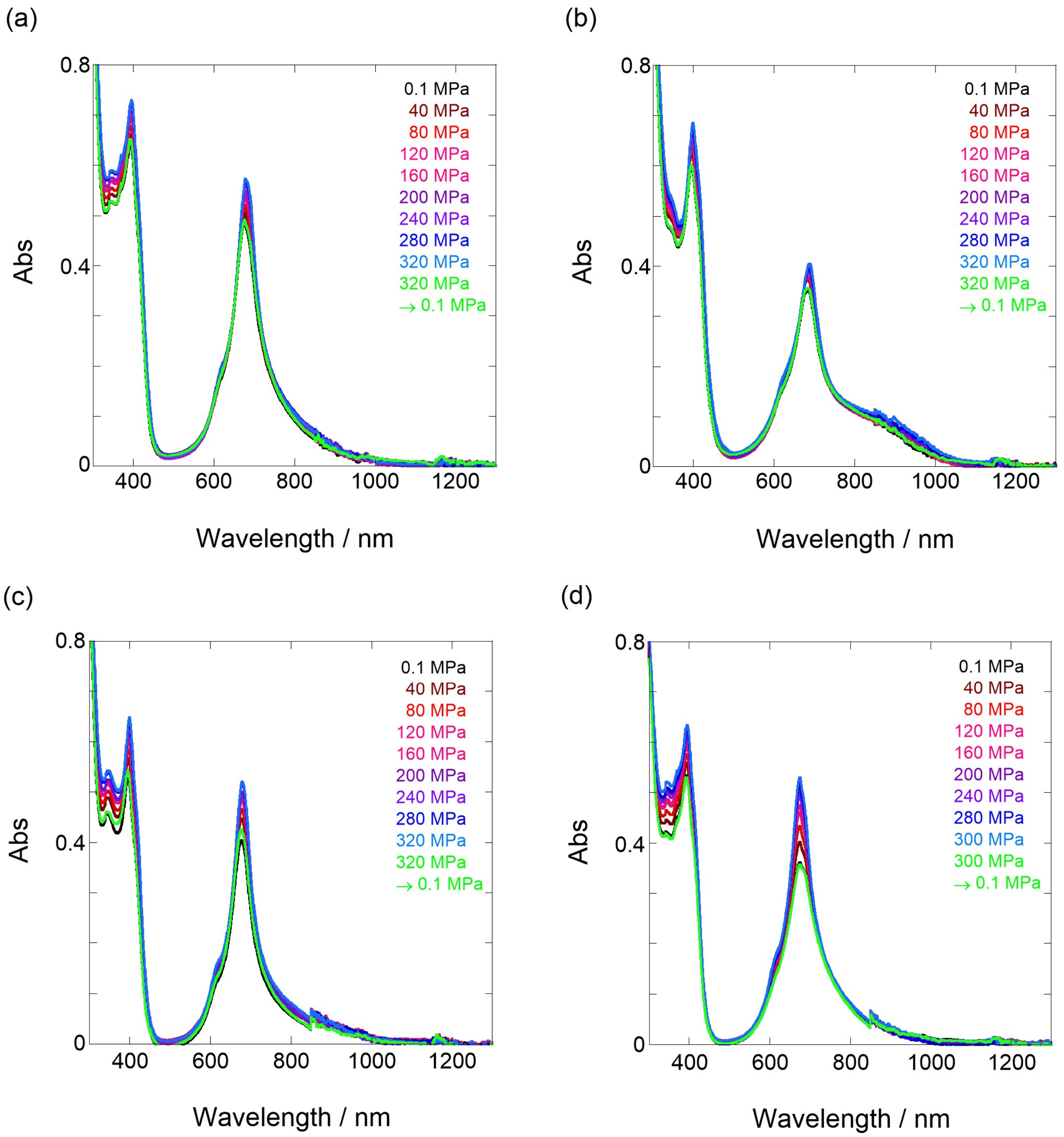

Figure S4. Pressure-dependent UV/vis/NIR spectra of **1Zn** obtained in (a) THF (33 μM), (b) toluene (33 μM), (c) dichloromethane (30 μM), and (d) ethyl acetate (36 μM) at 298 K using the high-pressure cell. Pressure applied: 0.1, 40, 80, 120, 160, 200, 240, 280, and 320 (300) MPa (from black to light blue). The green lines show the spectra recorded at 0.1 MPa after depressurization from 320 (300) MPa.

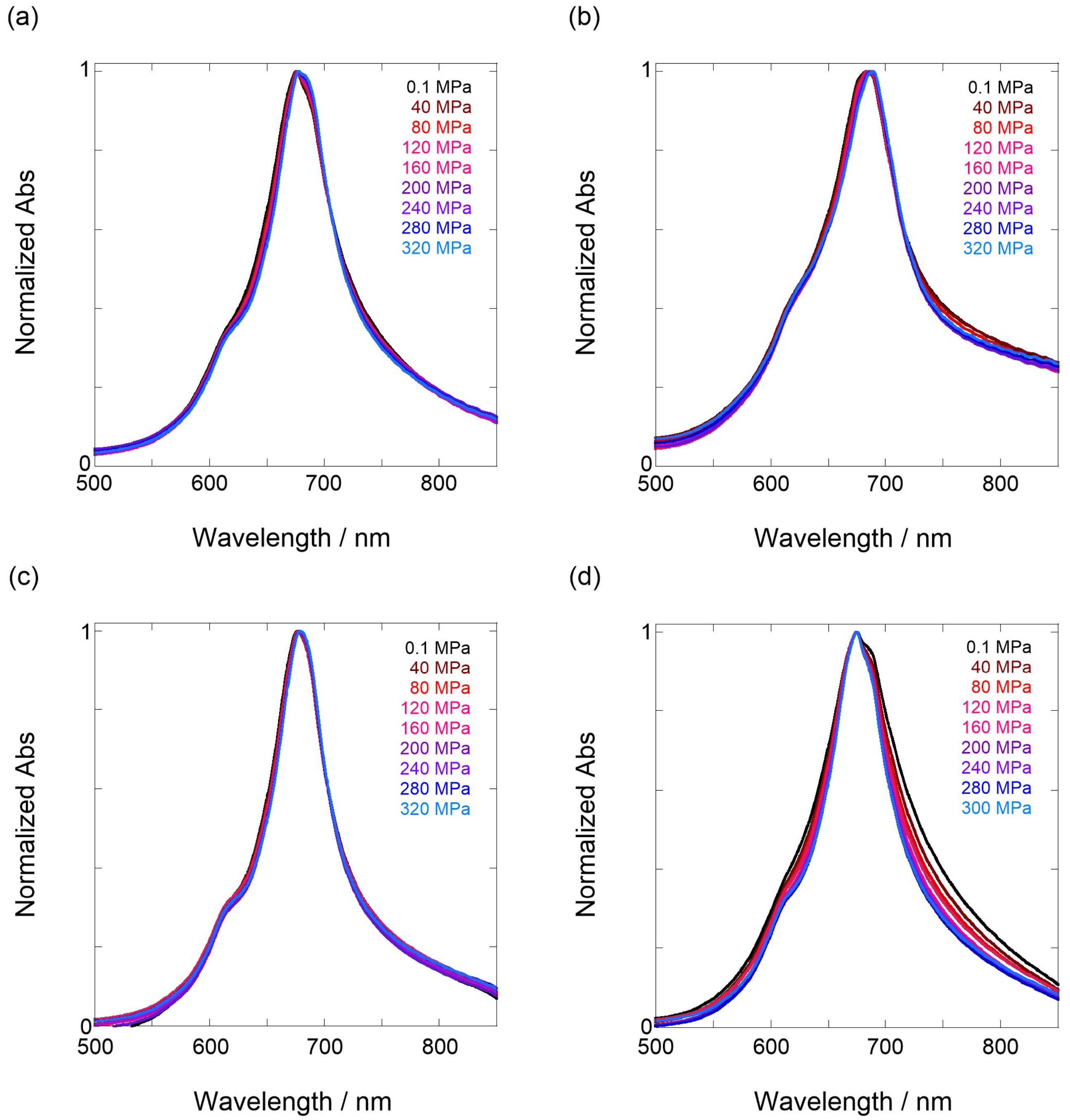

Figure S5. Normalized pressure-dependent UV/vis/NIR spectra of **1Zn** obtained in (a) THF (33 μM), (b) toluene (33 μM), (c) dichloromethane (30 μM), and (d) ethyl acetate (36 μM).

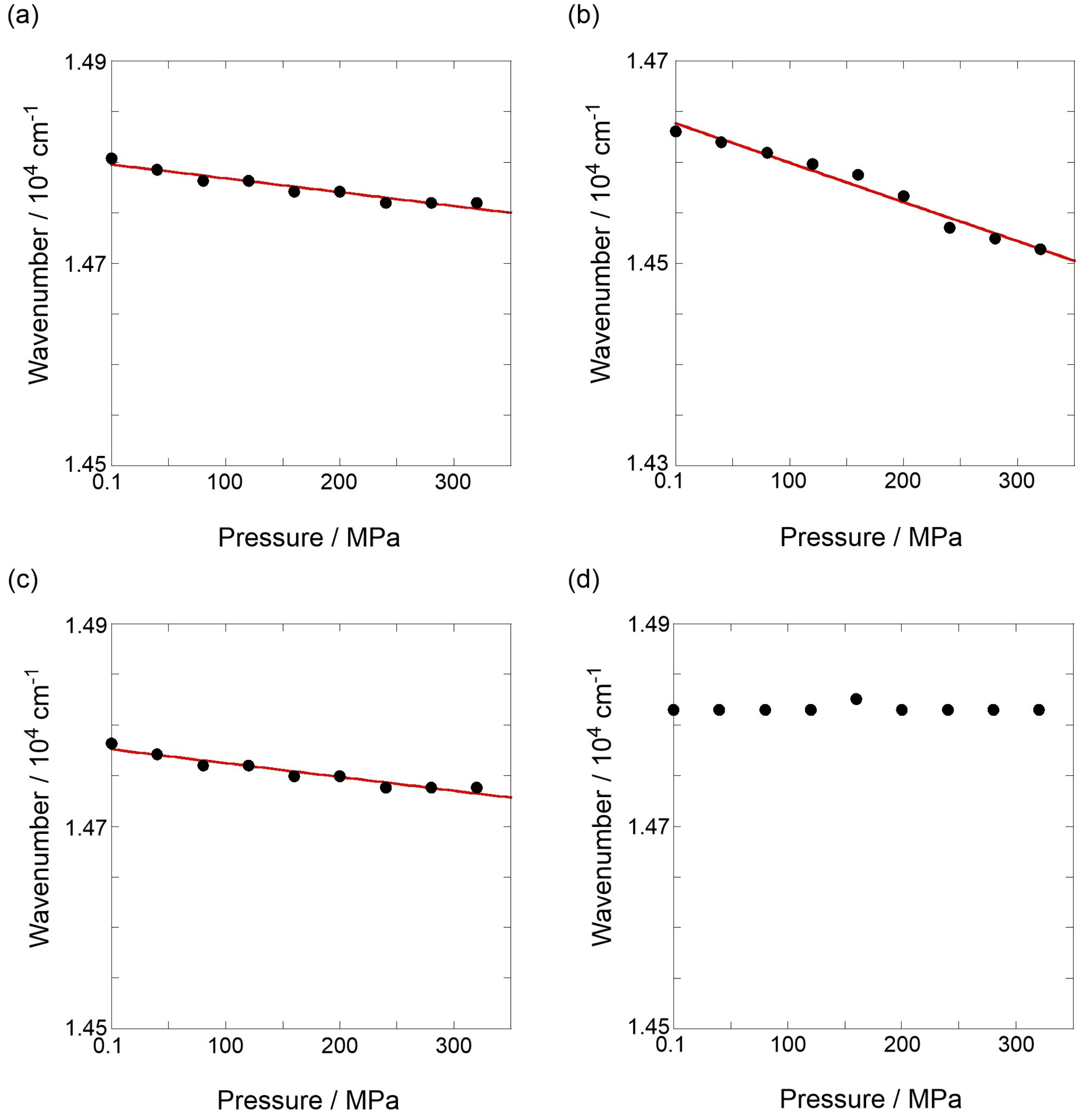

Figure S6. Wavenumber changes observed for the pressure-induced absorption maxima of **1Zn** in (a) THF (r = 0.961, slope: –0.14 cm^{-1} MPa^{-1}), (b) toluene (r = 0.986, slope: –0.39 cm^{-1} MPa^{-1}), (c) dichloromethane (r = 0.961, slope: –0.14 cm^{-1} MPa^{-1}), and (d) ethyl acetate (nearly flat).

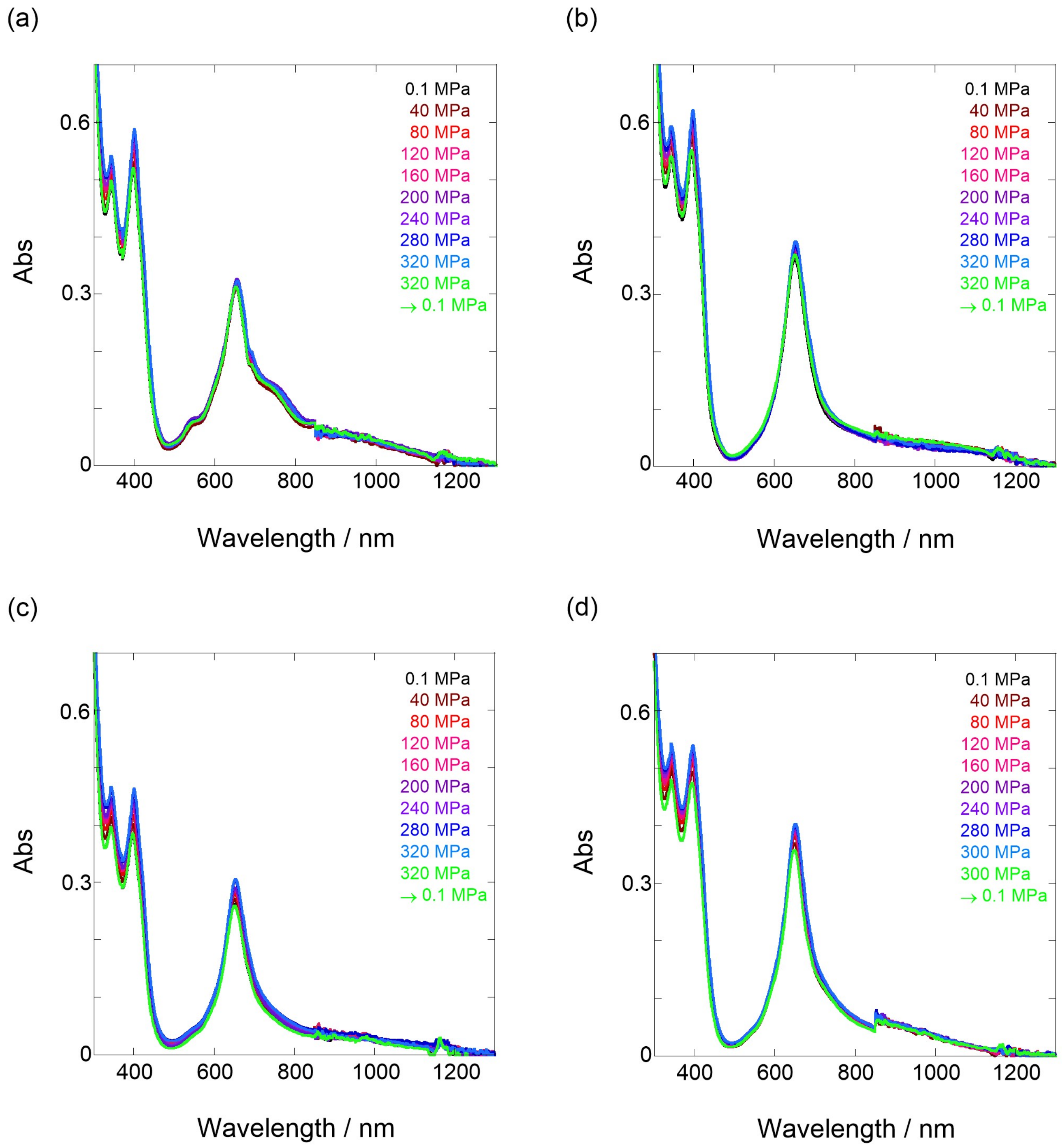

Figure S7. Pressure-dependent UV/vis/NIR spectra of **1Zn$_2$** obtained in (a) THF (13 µM), (b) toluene (14 µM), (c) dichloromethane (11 µM), and (d) ethyl acetate (14 µM) at 298 K using the high-pressure cell. Pressure applied: 0.1, 40, 80, 120, 160, 200, 240, 280, and 320 (300) MPa (from black to light blue). The green lines show the spectra recorded at 0.1 MPa after depressurization from 320 (300) MPa.

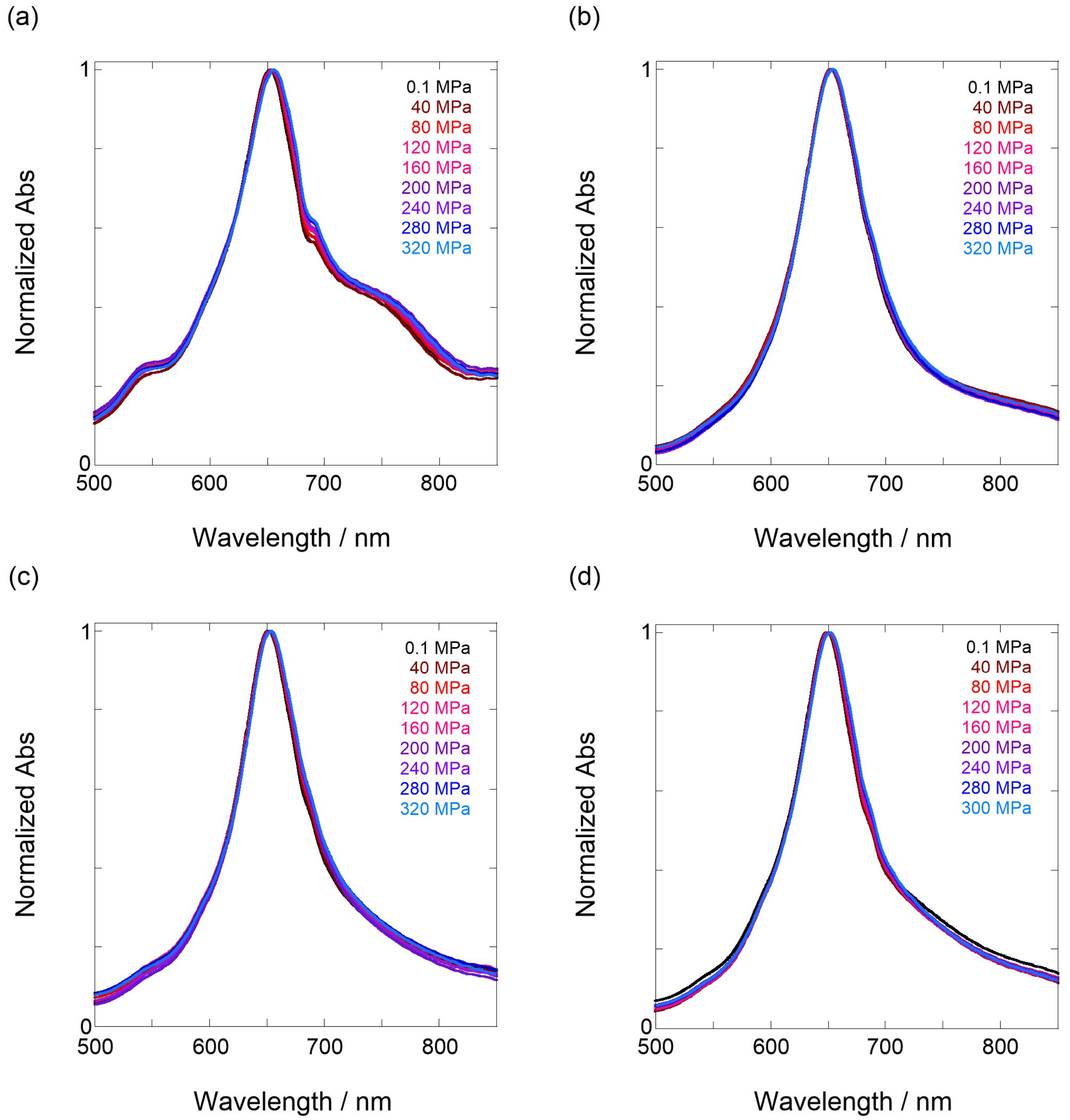

Figure S8. Normalized pressure-dependent UV/vis/NIR spectra of **1Zn₂** obtained in (a) THF (13 μM), (b) toluene (14 μM), (c) dichloromethane (11 μM), and (d) ethyl acetate (14 μM).

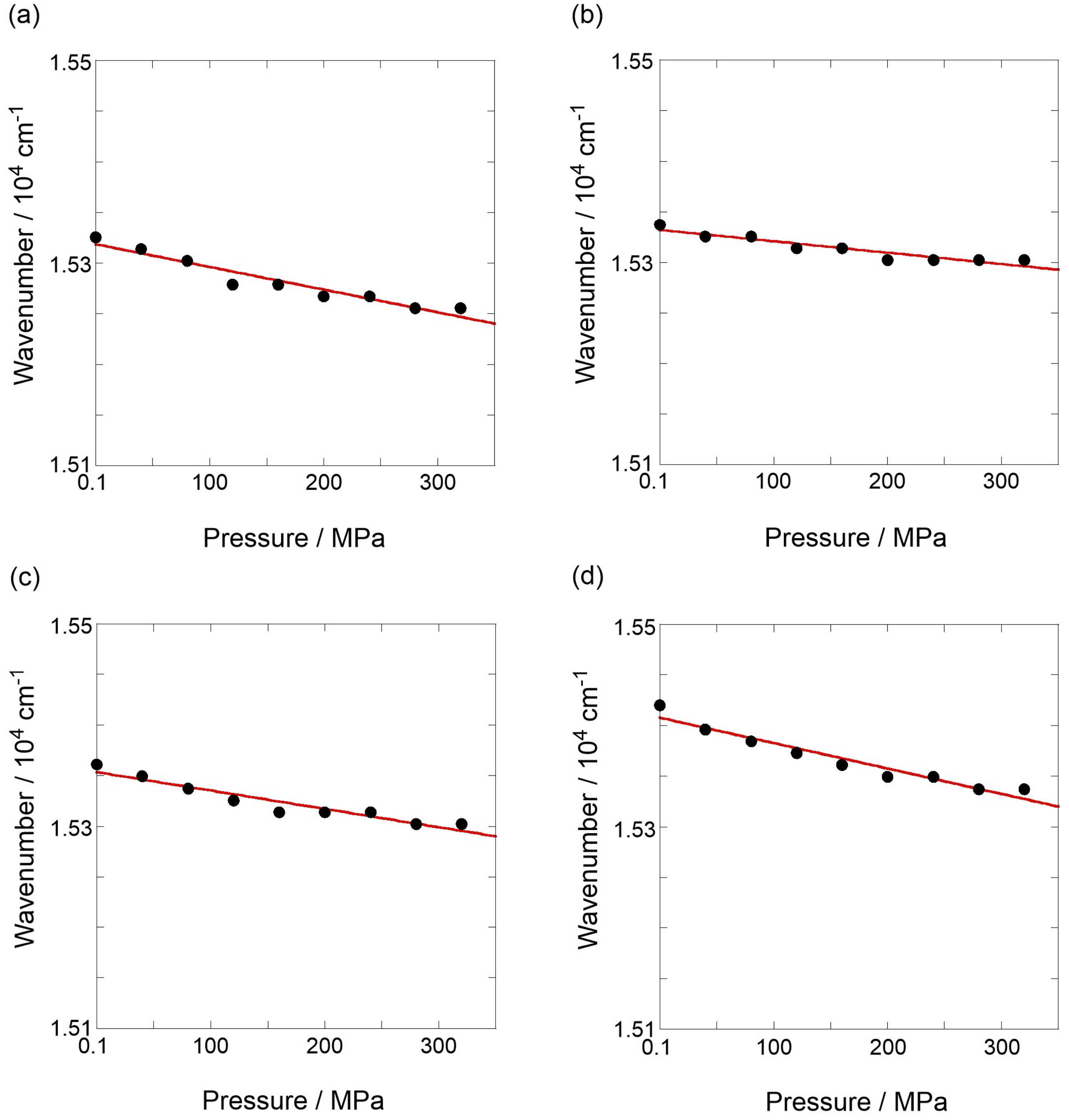

Figure S9. Wavenumber changes observed for the pressure-induced absorption maxima of **1Zn$_2$** in (a) THF (r = 0.963, slope: –0.22 cm^{-1} MPa^{-1}), (b) toluene (r = 0.939, slope: –0.11 cm^{-1} MPa^{-1}), (c) dichloromethane, (r = 0.957, slope: –0.18 cm^{-1} MPa^{-1}), and (d) ethyl acetate (r = 0.968, slope: –0.25 cm^{-1} MPa^{-1}).

Pressure-dependent kinetic studies

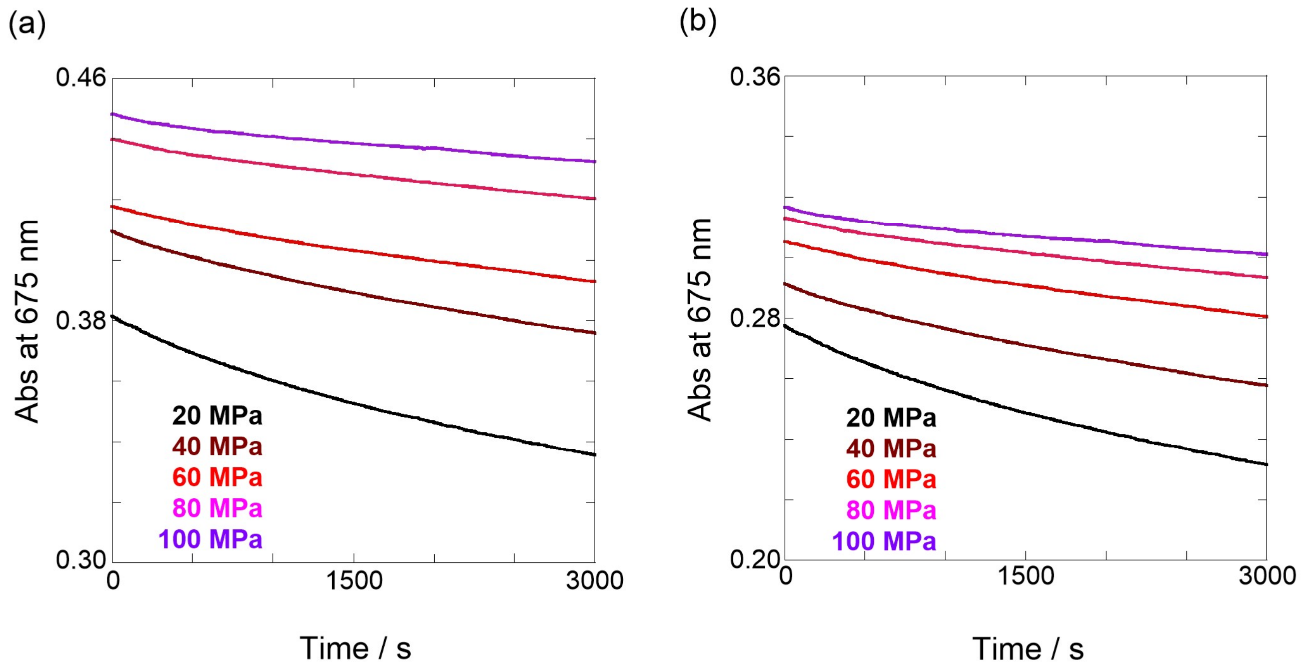

Figure S10. (a) Pressure-dependent UV/vis/NIR spectroscopic time-course curves of **1Zn** (29 μM) recorded during dimerization in ethyl acetate at a wavelength of 675 nm, temperature of 313 K, and pressures of 20, 40, 60, 80, and 100 MPa using the high-pressure cell. (b) Corrected spectra presented (a).

$Abs_{313K\_cal(t=0)}$ (estimated from Figures S11 and S12) and $Abs_{313K\_app(t=0)}$ (shown in Figure S10a) represent the monomer absorbance values at 313 K estimated by extrapolating the calibration curves and actual values, respectively.

$$\Delta Abs_{\ P} = Abs_{313K\_app(t=0)} - Abs_{313K\_cal(t=0)}$$

Absorbance at time = *t* ($Abs'_{app(t)}$) was corrected as follows:

$$Abs'_{app(t)} = Abs_{app(t)} - \Delta Abs_{\ P}$$

After that, we obtained the corrected spectra depicted in Figure S10b.

The ε_D values determined from Figures S13 and S14 were processed using Eq. (S6) to obtain Figure S15.

Finally, the fitting results presented in Figure S15 produced k_{dim} values at various pressures.

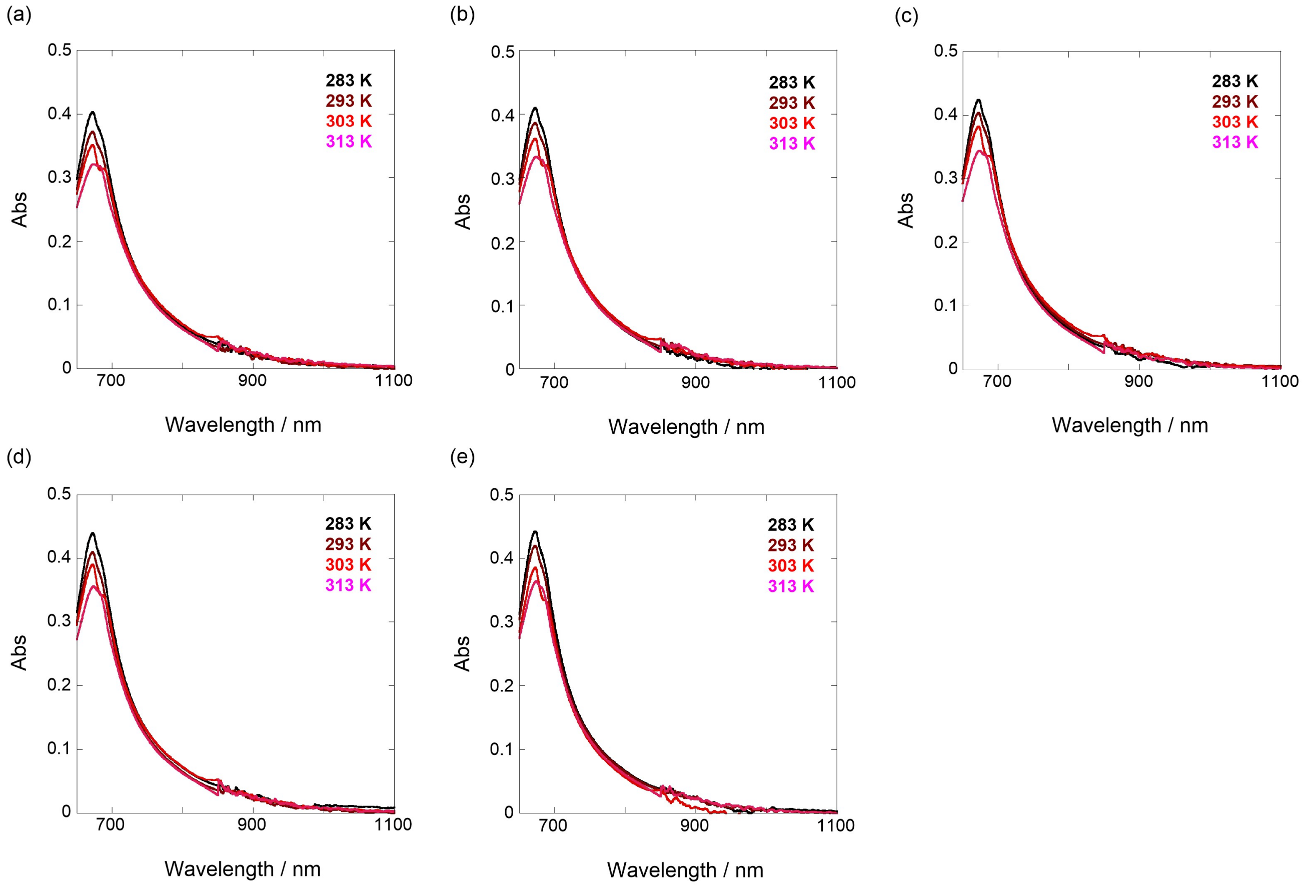

Figure S11. Temperature-dependent UV/vis/NIR spectra of **1Zn** (29 μM) obtained in ethyl acetate at (a) 20, (b) 40, (c) 60, (d) 80, and (e) 100 MPa using the high-pressure cell. Temperature applied: 283, 293, 303, and 313 K (from black to pink).

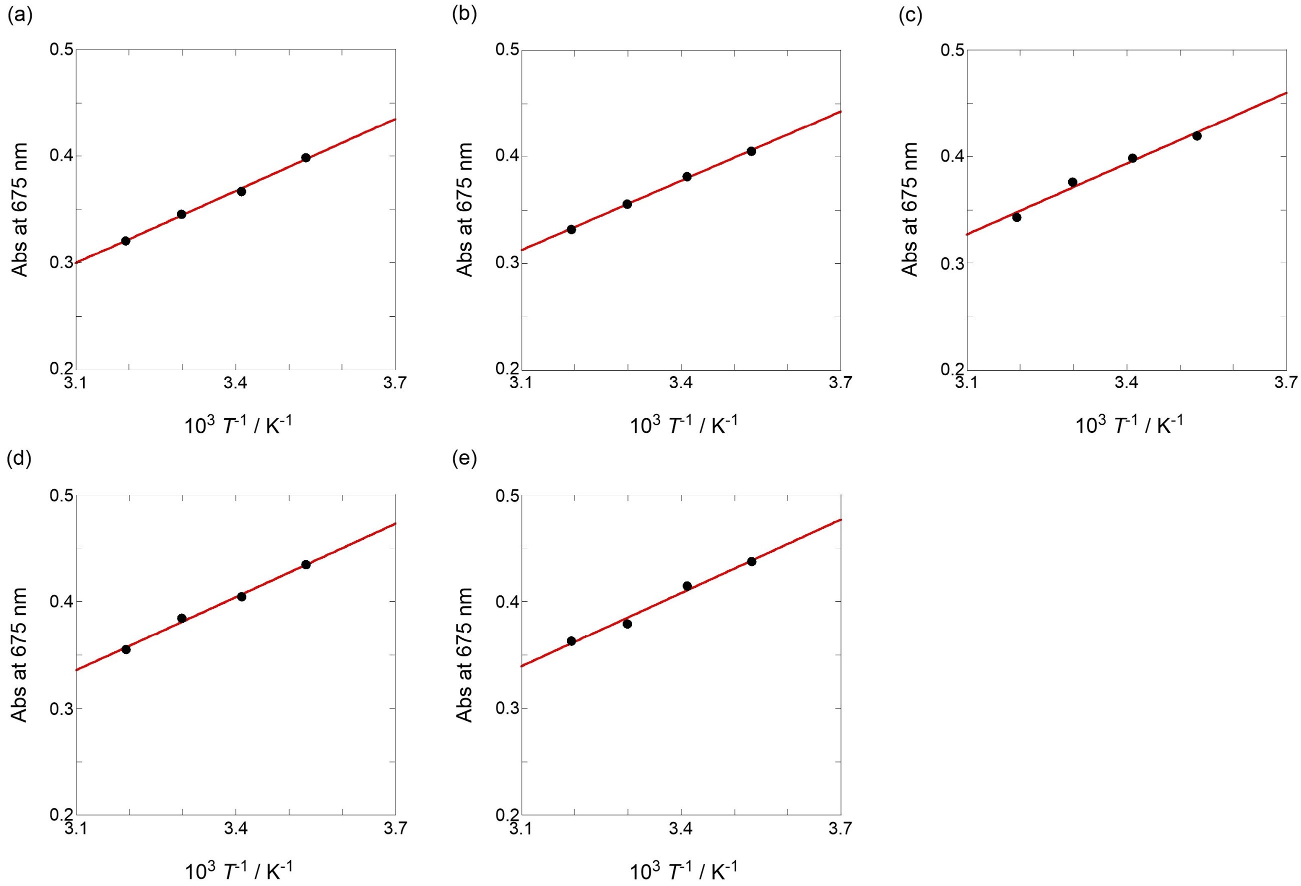

Figure S12. Temperature-dependent absorbance (at 675 nm) plots of **1Zn** (29 μM) obtained in ethyl acetate at (a) 20, (b) 40, (c) 60, (d) 80, and (e) 100 MPa.

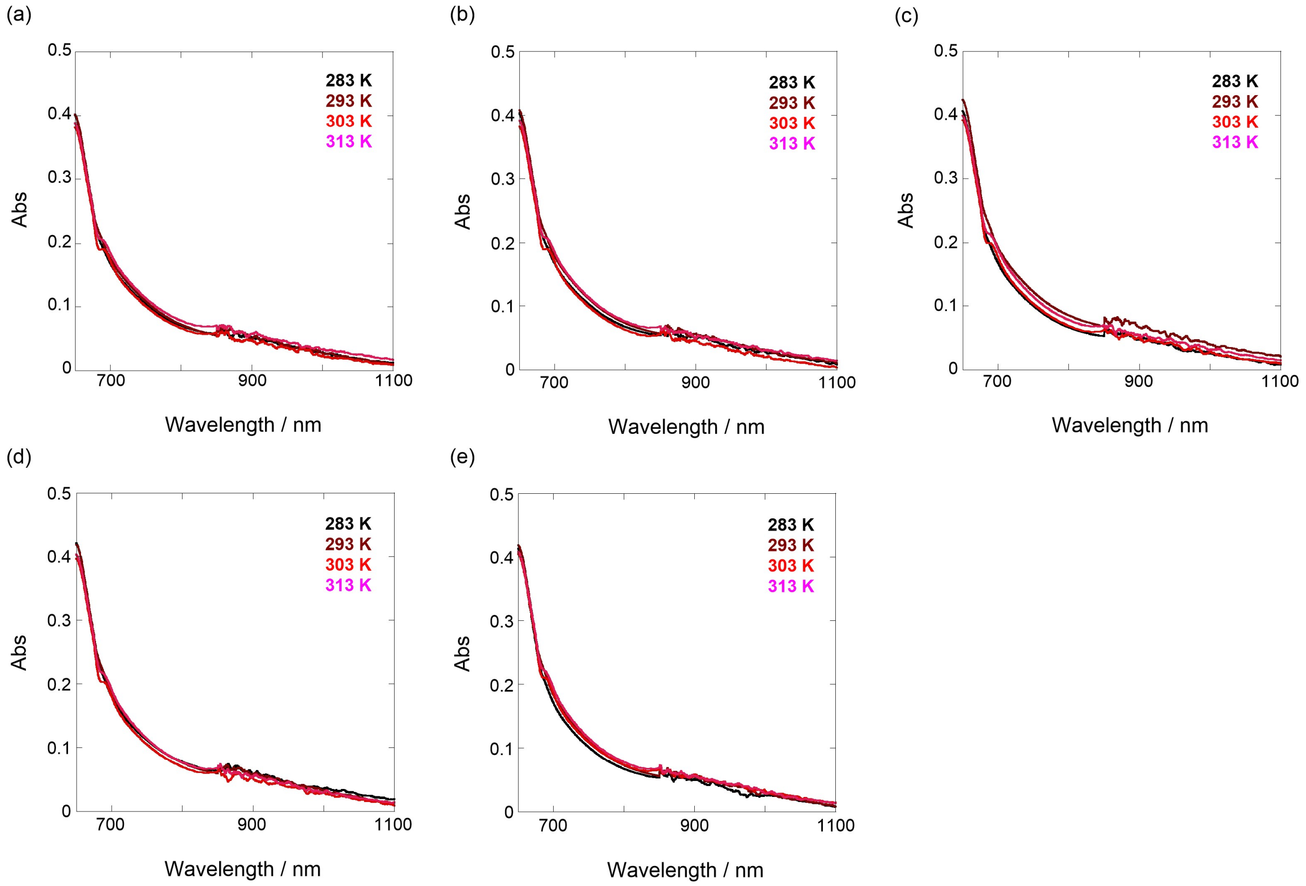

Figure S13. Temperature-dependent UV/vis/NIR spectra of **1Zn$_2$** (17 μM) obtained in ethyl acetate at (a) 20, (b) 40, (c) 60, (d) 80, and (e) 100 MPa using the high-pressure cell. Temperature applied: 283, 293, 303, and 313 K (from black to pink).

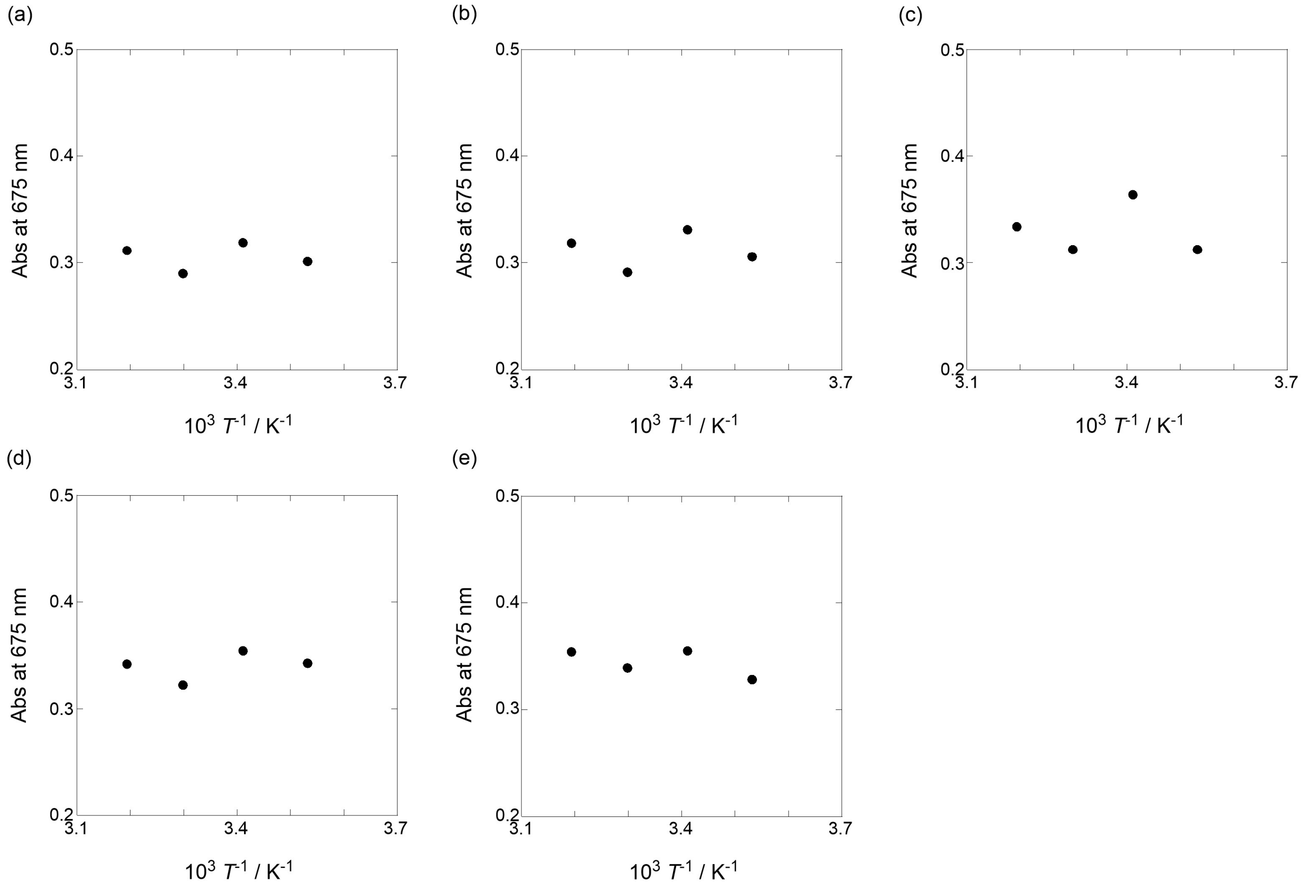

Figure S14. Temperature-dependent absorbance (at 675 nm) plots of **1Zn$_2$** (17 μM) obtained in ethyl acetate at (a) 20, (b) 40, (c) 60, (d) 80, and (e) 100 MPa.

Each ε_D magnitude was extracted from Figure S14 as an average value.

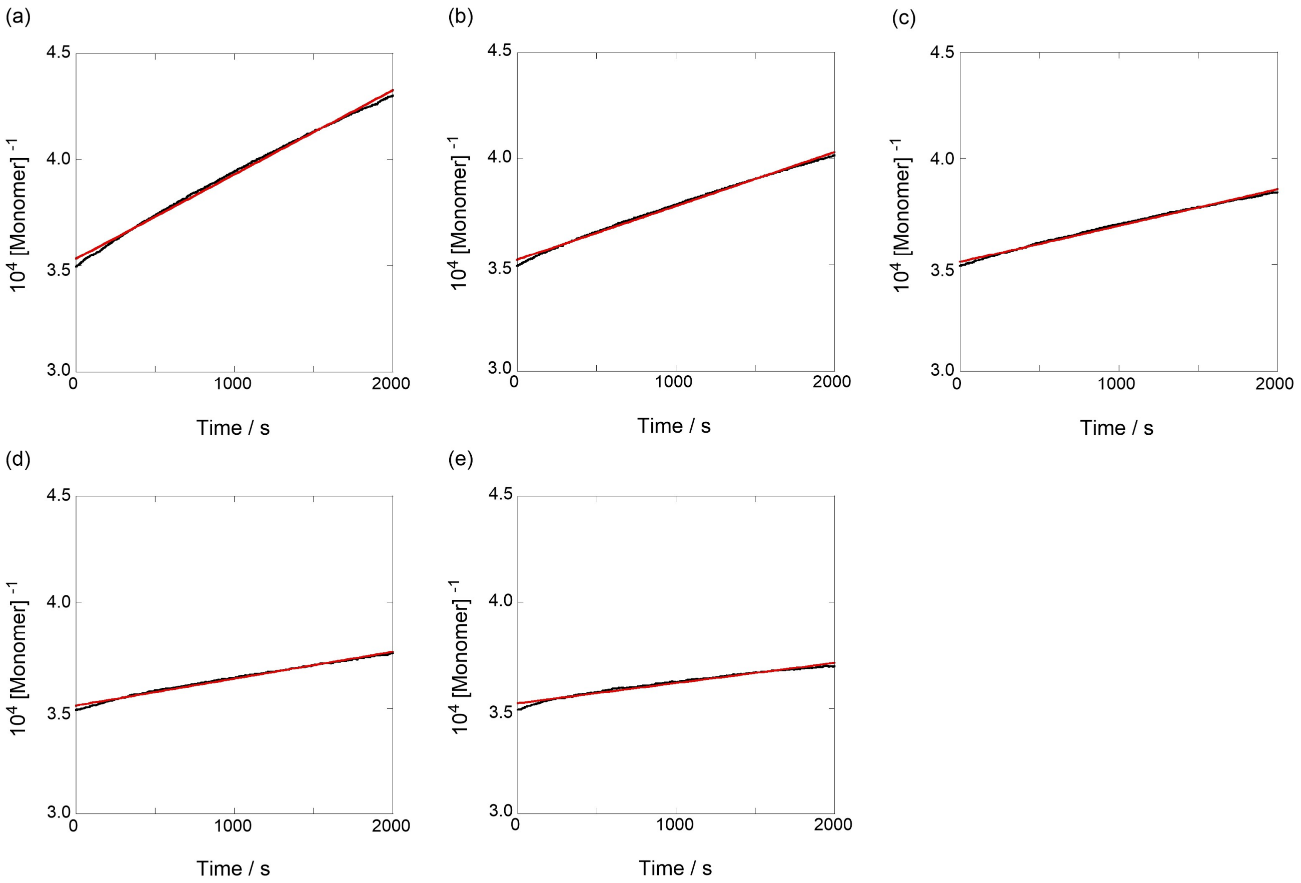

Figure S15. Pressure-dependent 1/[Monomer] plots obtained for ethyl acetate at a temperature of 333 K and pressures of (a) 20 MPa (r = 0.998), (b) 40 MPa (r = 0.998), (c) 60 MPa (r = 0.997), (d) 80 MPa (r = 0.996), and (e) 100 MPa (r = 0.989).

Table S2. Dimerization rates in ethyl acetate determined at a temperature of 333 K in the pressure range of 0.1–100 MPa

P / MPa	0.1	20	40	60	80	100
k_{dim} / M^{-1} s^{-1}	10.1	4.0	2.5	1.7	1.3	0.9

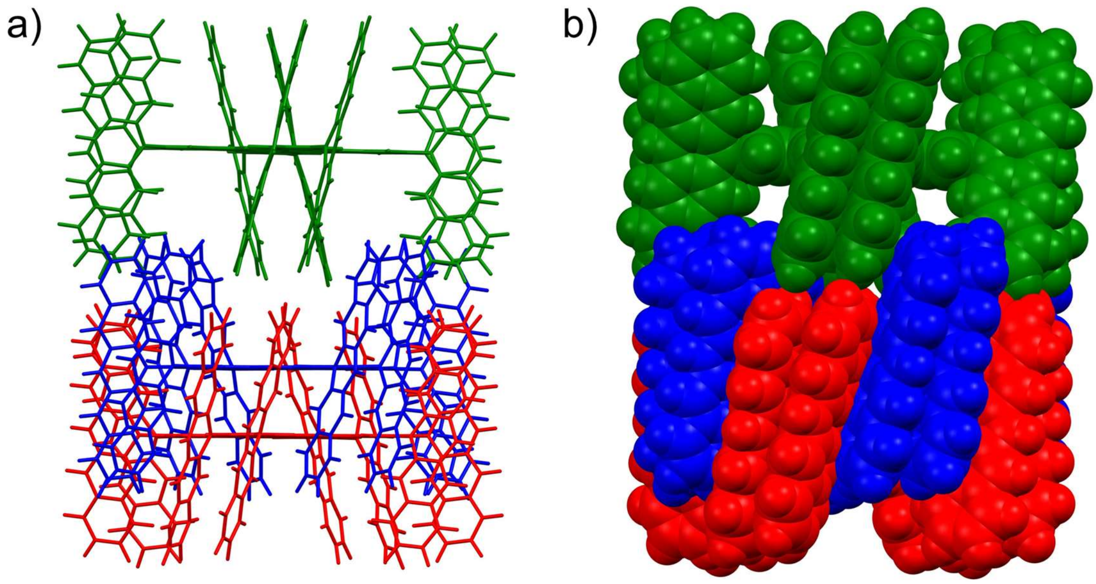

Figure S16. (a) Molecular model of **1Zn'3** (each monomer is shown in a different color) shown in a) wireframe model and (b) space filling model.

1Zn' (D_4) M06-2X/6-31G* (for C, H, and N) LANL2DZ (for Zn)

$E = -8760.41784319$ hartree

C	-1.119984	2.785701	0.000495
C	-0.700881	4.187260	0.002415
N	-1.412232	7.814289	0.029880
C	0.700881	4.187260	-0.002415
N	1.412232	7.814289	-0.029880
C	1.119984	2.785701	-0.000495
N	-2.387666	2.387666	0.000000
N	0.000000	2.007877	0.000000
C	1.900515	8.312210	-1.258831
C	1.304802	8.642260	1.109110
C	1.438218	10.057298	0.948560
C	2.011064	9.733677	-1.408556
N	1.722848	10.538053	-0.316384
C	1.259670	10.889646	2.026120
C	2.371141	10.271183	-2.617728
H	1.804020	11.536621	-0.437867
C	-1.900515	8.312210	1.258831
C	-1.304802	8.642260	-1.109110

C	-1.438218	10.057298	-0.948560
C	-2.011064	9.733677	1.408556
N	-1.722848	10.538053	0.316384
C	-1.259670	10.889646	-2.026120
C	-2.371141	10.271183	2.617728
H	-1.804020	11.536621	0.437867
C	0.929209	10.379133	3.307434
H	1.351933	11.965449	1.889769
C	0.681806	11.220606	4.420908
C	0.824434	8.972737	3.474868
C	1.042523	8.129542	2.353027
H	0.950086	7.054926	2.482728
C	0.348018	10.687712	5.642533
H	0.759261	12.297441	4.290620
H	0.152204	11.343923	6.485397
C	0.249502	9.287610	5.809929
C	0.484986	8.451100	4.746719
H	-0.033723	8.877283	6.773831
H	0.390060	7.374034	4.861076
C	2.644963	9.452147	-3.741425
H	2.416260	11.352711	-2.727892
C	2.984956	9.990533	-5.005491
C	2.564413	8.043152	-3.589261
C	2.193656	7.504362	-2.327582
H	2.099993	6.427314	-2.236614
C	3.231363	9.165751	-6.076371
H	3.035134	11.070785	-5.116429
H	3.487648	9.590125	-7.042383
C	3.149928	7.762917	-5.927024
C	2.825046	7.215834	-4.708621
H	3.349507	7.118110	-6.777375
H	2.761739	6.137103	-4.587041
C	-0.929209	10.379133	-3.307434
H	-1.351933	11.965449	-1.889769
C	-0.824434	8.972737	-3.474868
C	-1.042523	8.129542	-2.353027
C	-0.484986	8.451100	-4.746719
H	-0.950086	7.054926	-2.482728

C	-2.644963	9.452147	3.741425
H	-2.416260	11.352711	2.727892
C	-2.564413	8.043152	3.589261
C	-2.193656	7.504362	2.327582
C	-2.825046	7.215834	4.708621
H	-2.099993	6.427314	2.236614
C	-0.249502	9.287610	-5.809929
H	-0.390060	7.374034	-4.861076
H	0.033723	8.877283	-6.773831
C	-0.348018	10.687712	-5.642533
C	-0.681806	11.220606	-4.420908
H	-0.152204	11.343923	-6.485397
H	-0.759261	12.297441	-4.290620
C	-3.149928	7.762917	5.927024
H	-2.761739	6.137103	4.587041
H	-3.349507	7.118110	6.777375
C	-3.231363	9.165751	6.076371
C	-2.984956	9.990533	5.005491
H	-3.487648	9.590125	7.042383
H	-3.035134	11.070785	5.116429
C	1.421204	5.379283	-0.003713
C	0.714573	6.575620	-0.009225
H	2.506820	5.388464	0.008970
C	-0.714573	6.575620	0.009225
C	-1.421204	5.379283	0.003713
H	-2.506820	5.388464	-0.008970
C	2.785701	1.119984	0.000495
C	4.187260	0.700881	0.002415
N	7.814289	1.412232	0.029880
C	4.187260	-0.700881	-0.002415
N	7.814289	-1.412232	-0.029880
C	2.785701	-1.119984	-0.000495
N	2.387666	2.387666	0.000000
N	2.007877	0.000000	0.000000
C	8.312210	-1.900515	-1.258831
C	8.642260	-1.304802	1.109110
C	10.057298	-1.438218	0.948560
C	9.733677	-2.011064	-1.408556

N	10.538053	-1.722848	-0.316384
C	10.889646	-1.259670	2.026120
C	10.271183	-2.371141	-2.617728
H	11.536621	-1.804020	-0.437867
C	8.312210	1.900515	1.258831
C	8.642260	1.304802	-1.109110
C	10.057298	1.438218	-0.948560
C	9.733677	2.011064	1.408556
N	10.538053	1.722848	0.316384
C	10.889646	1.259670	-2.026120
C	10.271183	2.371141	2.617728
H	11.536621	1.804020	0.437867
C	10.379133	-0.929209	3.307434
H	11.965449	-1.351933	1.889769
C	11.220606	-0.681806	4.420908
C	8.972737	-0.824434	3.474868
C	8.129542	-1.042523	2.353027
H	7.054926	-0.950086	2.482728
C	10.687712	-0.348018	5.642533
H	12.297441	-0.759261	4.290620
H	11.343923	-0.152204	6.485397
C	9.287610	-0.249502	5.809929
C	8.451100	-0.484986	4.746719
H	8.877283	0.033723	6.773831
H	7.374034	-0.390060	4.861076
C	9.452147	-2.644963	-3.741425
H	11.352711	-2.416260	-2.727892
C	9.990533	-2.984956	-5.005491
C	8.043152	-2.564413	-3.589261
C	7.504362	-2.193656	-2.327582
H	6.427314	-2.099993	-2.236614
C	9.165751	-3.231363	-6.076371
H	11.070785	-3.035134	-5.116429
H	9.590125	-3.487648	-7.042383
C	7.762917	-3.149928	-5.927024
C	7.215834	-2.825046	-4.708621
H	7.118110	-3.349507	-6.777375
H	6.137103	-2.761739	-4.587041

C	10.379133	0.929209	-3.307434
H	11.965449	1.351933	-1.889769
C	8.972737	0.824434	-3.474868
C	8.129542	1.042523	-2.353027
C	8.451100	0.484986	-4.746719
H	7.054926	0.950086	-2.482728
C	9.452147	2.644963	3.741425
H	11.352711	2.416260	2.727892
C	8.043152	2.564413	3.589261
C	7.504362	2.193656	2.327582
C	7.215834	2.825046	4.708621
H	6.427314	2.099993	2.236614
C	9.287610	0.249502	-5.809929
H	7.374034	0.390060	-4.861076
H	8.877283	-0.033723	-6.773831
C	10.687712	0.348018	-5.642533
C	11.220606	0.681806	-4.420908
H	11.343923	0.152204	-6.485397
H	12.297441	0.759261	-4.290620
C	7.762917	3.149928	5.927024
H	6.137103	2.761739	4.587041
H	7.118110	3.349507	6.777375
C	9.165751	3.231363	6.076371
C	9.990533	2.984956	5.005491
H	9.590125	3.487648	7.042383
H	11.070785	3.035134	5.116429
C	5.379283	-1.421204	-0.003713
C	6.575620	-0.714573	-0.009225
H	5.388464	-2.506820	0.008970
C	6.575620	0.714573	0.009225
C	5.379283	1.421204	0.003713
H	5.388464	2.506820	-0.008970
C	1.119984	-2.785701	0.000495
C	0.700881	-4.187260	0.002415
N	1.412232	-7.814289	0.029880
C	-0.700881	-4.187260	-0.002415
N	-1.412232	-7.814289	-0.029880
C	-1.119984	-2.785701	-0.000495

N	2.387666	-2.387666	0.000000
N	0.000000	-2.007877	0.000000
C	-1.900515	-8.312210	-1.258831
C	-1.304802	-8.642260	1.109110
C	-1.438218	-10.057290	0.948560
C	-2.011064	-9.733677	-1.408556
N	-1.722848	-10.538050	-0.316384
C	-1.259670	-10.889640	2.026120
C	-2.371141	-10.271180	-2.617728
H	-1.804020	-11.536620	-0.437867
C	1.900515	-8.312210	1.258831
C	1.304802	-8.642260	-1.109110
C	1.438218	-10.057290	-0.948560
C	2.011064	-9.733677	1.408556
N	1.722848	-10.538050	0.316384
C	1.259670	-10.889640	-2.026120
C	2.371141	-10.271180	2.617728
H	1.804020	-11.536620	0.437867
C	-0.929209	-10.379130	3.307434
H	-1.351933	-11.965440	1.889769
C	-0.681806	-11.220600	4.420908
C	-0.824434	-8.972737	3.474868
C	-1.042523	-8.129542	2.353027
H	-0.950086	-7.054926	2.482728
C	-0.348018	-10.687710	5.642533
H	-0.759261	-12.297440	4.290620
H	-0.152204	-11.343920	6.485397
C	-0.249502	-9.287610	5.809929
C	-0.484986	-8.451100	4.746719
H	0.033723	-8.877283	6.773831
H	-0.390060	-7.374034	4.861076
C	-2.644963	-9.452147	-3.741425
H	-2.416260	-11.352710	-2.727892
C	-2.984956	-9.990533	-5.005491
C	-2.564413	-8.043152	-3.589261
C	-2.193656	-7.504362	-2.327582
H	-2.099993	-6.427314	-2.236614
C	-3.231363	-9.165751	-6.076371

H	-3.035134	-11.070780	-5.116429
H	-3.487648	-9.590125	-7.042383
C	-3.149928	-7.762917	-5.927024
C	-2.825046	-7.215834	-4.708621
H	-3.349507	-7.118110	-6.777375
H	-2.761739	-6.137103	-4.587041
C	0.929209	-10.379130	-3.307434
H	1.351933	-11.965440	-1.889769
C	0.824434	-8.972737	-3.474868
C	1.042523	-8.129542	-2.353027
C	0.484986	-8.451100	-4.746719
H	0.950086	-7.054926	-2.482728
C	2.644963	-9.452147	3.741425
H	2.416260	-11.352710	2.727892
C	2.564413	-8.043152	3.589261
C	2.193656	-7.504362	2.327582
C	2.825046	-7.215834	4.708621
H	2.099993	-6.427314	2.236614
C	0.249502	-9.287610	-5.809929
H	0.390060	-7.374034	-4.861076
H	-0.033723	-8.877283	-6.773831
C	0.348018	-10.687710	-5.642533
C	0.681806	-11.220600	-4.420908
H	0.152204	-11.343920	-6.485397
H	0.759261	-12.297440	-4.290620
C	3.149928	-7.762917	5.927024
H	2.761739	-6.137103	4.587041
H	3.349507	-7.118110	6.777375
C	3.231363	-9.165751	6.076371
C	2.984956	-9.990533	5.005491
H	3.487648	-9.590125	7.042383
H	3.035134	-11.070780	5.116429
C	-1.421204	-5.379283	-0.003713
C	-0.714573	-6.575620	-0.009225
H	-2.506820	-5.388464	0.008970
C	0.714573	-6.575620	0.009225
C	1.421204	-5.379283	0.003713
H	2.506820	-5.388464	-0.008970

C	-2.785701	-1.119984	0.000495
C	-4.187260	-0.700881	0.002415
N	-7.814289	-1.412232	0.029880
C	-4.187260	0.700881	-0.002415
N	-7.814289	1.412232	-0.029880
C	-2.785701	1.119984	-0.000495
N	-2.387666	-2.387666	0.000000
N	-2.007877	0.000000	0.000000
C	-8.312210	1.900515	-1.258831
C	-8.642260	1.304802	1.109110
C	-10.057290	1.438218	0.948560
C	-9.733677	2.011064	-1.408556
N	-10.538050	1.722848	-0.316384
C	-10.889640	1.259670	2.026120
C	-10.271180	2.371141	-2.617728
H	-11.536620	1.804020	-0.437867
C	-8.312210	-1.900515	1.258831
C	-8.642260	-1.304802	-1.109110
C	-10.057290	-1.438218	-0.948560
C	-9.733677	-2.011064	1.408556
N	-10.538050	-1.722848	0.316384
C	-10.889640	-1.259670	-2.026120
C	-10.271180	-2.371141	2.617728
H	-11.536620	-1.804020	0.437867
C	-10.379130	0.929209	3.307434
H	-11.965440	1.351933	1.889769
C	-11.220600	0.681806	4.420908
C	-8.972737	0.824434	3.474868
C	-8.129542	1.042523	2.353027
H	-7.054926	0.950086	2.482728
C	-10.687710	0.348018	5.642533
H	-12.297440	0.759261	4.290620
H	-11.343920	0.152204	6.485397
C	-9.287610	0.249502	5.809929
C	-8.451100	0.484986	4.746719
H	-8.877283	-0.033723	6.773831
H	-7.374034	0.390060	4.861076
C	-9.452147	2.644963	-3.741425

H	-11.352710	2.416260	-2.727892
C	-9.990533	2.984956	-5.005491
C	-8.043152	2.564413	-3.589261
C	-7.504362	2.193656	-2.327582
H	-6.427314	2.099993	-2.236614
C	-9.165751	3.231363	-6.076371
H	-11.070780	3.035134	-5.116429
H	-9.590125	3.487648	-7.042383
C	-7.762917	3.149928	-5.927024
C	-7.215834	2.825046	-4.708621
H	-7.118110	3.349507	-6.777375
H	-6.137103	2.761739	-4.587041
C	-10.379130	-0.929209	-3.307434
H	-11.965440	-1.351933	-1.889769
C	-8.972737	-0.824434	-3.474868
C	-8.129542	-1.042523	-2.353027
C	-8.451100	-0.484986	-4.746719
H	-7.054926	-0.950086	-2.482728
C	-9.452147	-2.644963	3.741425
H	-11.352710	-2.416260	2.727892
C	-8.043152	-2.564413	3.589261
C	-7.504362	-2.193656	2.327582
C	-7.215834	-2.825046	4.708621
H	-6.427314	-2.099993	2.236614
C	-9.287610	-0.249502	-5.809929
H	-7.374034	-0.390060	-4.861076
H	-8.877283	0.033723	-6.773831
C	-10.687710	-0.348018	-5.642533
C	-11.220600	-0.681806	-4.420908
H	-11.343920	-0.152204	-6.485397
H	-12.297440	-0.759261	-4.290620
C	-7.762917	-3.149928	5.927024
H	-6.137103	-2.761739	4.587041
H	-7.118110	-3.349507	6.777375
C	-9.165751	-3.231363	6.076371
C	-9.990533	-2.984956	5.005491
H	-9.590125	-3.487648	7.042383
H	-11.070780	-3.035134	5.116429

C	-5.379283	1.421204	-0.003713
C	-6.575620	0.714573	-0.009225
H	-5.388464	2.506820	0.008970
C	-6.575620	-0.714573	0.009225
C	-5.379283	-1.421204	0.003713
H	-5.388464	-2.506820	-0.008970
Z	0.000000	0.000000	0.000000

1Zn'2 (D_4) M06-2X/6-31G* (for C, H, and N) LANL2DZ (for Zn)

$E = -17520.9877471$ hartree

C	1.930277	2.304499	1.570278
C	1.963658	3.767678	1.564064
N	3.732438	7.014457	1.837075
C	0.630413	4.196701	1.589538
N	1.054370	7.855320	1.858820
C	-0.201784	2.994672	1.582425
N	3.011094	1.531417	1.579821
N	0.623187	1.907832	1.577230
C	0.528050	8.313663	3.086447
C	1.433585	8.747724	0.832706
C	1.384734	10.155404	1.084693
C	0.505671	9.727680	3.323311
N	0.966108	10.580202	2.331285
C	1.761565	11.044033	0.107395
C	0.067693	10.220006	4.525266
H	0.756285	11.561769	2.441618
C	4.480163	7.504964	0.741638
C	3.740728	7.693725	3.076344
C	4.136632	9.068869	3.101869
C	4.960129	8.853077	0.815837
N	4.631666	9.609114	1.931171
C	3.992047	9.802143	4.252770
C	5.676323	9.387841	-0.223424
H	4.932981	10.572380	1.945067
C	2.219906	10.605577	-1.162244
H	1.717952	12.110825	0.319070

C	2.647908	11.498335	-2.177274
C	2.259034	9.210502	-1.420325
C	1.826828	8.314269	-0.406607
H	1.861156	7.251284	-0.616590
C	3.108064	11.021876	-3.382838
H	2.619298	12.567957	-1.980447
H	3.438479	11.716835	-4.149561
C	3.157980	9.631793	-3.634970
C	2.735398	8.748693	-2.672020
H	3.521628	9.263314	-4.589022
H	2.763643	7.677953	-2.859274
C	-0.392349	9.364683	5.555540
H	0.071230	11.295228	4.689855
C	-0.843563	9.852208	6.806088
C	-0.397401	7.966273	5.318292
C	0.079066	7.472966	4.075189
H	0.107676	6.398064	3.937384
C	-1.283020	8.987854	7.780132
H	-0.832098	10.924871	6.983223
H	-1.627059	9.373381	8.735018
C	-1.296342	7.594766	7.541557
C	-0.861865	7.098150	6.336147
H	-1.652261	6.918957	8.313085
H	-0.865700	6.027129	6.146797
C	3.463871	9.222402	5.435858
H	4.264201	10.855403	4.252586
C	3.141408	7.838931	5.433425
C	3.314590	7.094849	4.233116
C	2.600905	7.256012	6.603693
H	3.024826	6.047292	4.220987
C	5.939307	8.644071	-1.400244
H	6.025971	10.415646	-0.156690
C	5.429633	7.322872	-1.498673
C	4.694678	6.787458	-0.404525
C	5.674492	6.584014	-2.682019
H	4.278420	5.788911	-0.490453
C	2.378220	8.011270	7.729899
H	2.336309	6.201166	6.587088

H	1.935497	7.558279	8.611068
C	2.696557	9.387323	7.731530
C	3.231347	9.977440	6.611397
H	2.505764	9.980584	8.620610
H	3.472694	11.037521	6.607459
C	6.404992	7.122037	-3.715191
H	5.287050	5.572400	-2.768824
H	6.600267	6.531295	-4.605332
C	6.906563	8.439579	-3.615132
C	6.674396	9.183095	-2.483626
H	7.476021	8.862277	-4.437221
H	7.049884	10.200036	-2.403272
C	0.310676	5.549396	1.642569
C	1.346283	6.475153	1.704400
H	-0.722570	5.879665	1.675825
C	2.707809	6.041718	1.672236
C	3.011267	4.685549	1.586349
H	4.042557	4.350158	1.604506
C	-2.304499	1.930277	1.570278
C	-3.767678	1.963658	1.564064
N	-7.014457	3.732438	1.837075
C	-4.196701	0.630413	1.589538
N	-7.855320	1.054370	1.858820
C	-2.994672	-0.201784	1.582425
N	-1.531417	3.011094	1.579821
N	-1.907832	0.623187	1.577230
C	-8.313663	0.528050	3.086447
C	-8.747724	1.433585	0.832706
C	-10.155404	1.384734	1.084693
C	-9.727680	0.505671	3.323311
N	-10.580202	0.966108	2.331285
C	-11.044033	1.761565	0.107395
C	-10.220006	0.067693	4.525266
H	-11.561769	0.756285	2.441618
C	-7.504964	4.480163	0.741638
C	-7.693725	3.740728	3.076344
C	-9.068869	4.136632	3.101869
C	-8.853077	4.960129	0.815837

N	-9.609114	4.631666	1.931171
C	-9.802143	3.992047	4.252770
C	-9.387841	5.676323	-0.223424
H	-10.572380	4.932981	1.945067
C	-10.605577	2.219906	-1.162244
H	-12.110825	1.717952	0.319070
C	-11.498335	2.647908	-2.177274
C	-9.210502	2.259034	-1.420325
C	-8.314269	1.826828	-0.406607
H	-7.251284	1.861156	-0.616590
C	-11.021876	3.108064	-3.382838
H	-12.567957	2.619298	-1.980447
H	-11.716835	3.438479	-4.149561
C	-9.631793	3.157980	-3.634970
C	-8.748693	2.735398	-2.672020
H	-9.263314	3.521628	-4.589022
H	-7.677953	2.763643	-2.859274
C	-9.364683	-0.392349	5.555540
H	-11.295228	0.071230	4.689855
C	-9.852208	-0.843563	6.806088
C	-7.966273	-0.397401	5.318292
C	-7.472966	0.079066	4.075189
H	-6.398064	0.107676	3.937384
C	-8.987854	-1.283020	7.780132
H	-10.924871	-0.832098	6.983223
H	-9.373381	-1.627059	8.735018
C	-7.594766	-1.296342	7.541557
C	-7.098150	-0.861865	6.336147
H	-6.918957	-1.652261	8.313085
H	-6.027129	-0.865700	6.146797
C	-9.222402	3.463871	5.435858
H	-10.855403	4.264201	4.252586
C	-7.838931	3.141408	5.433425
C	-7.094849	3.314590	4.233116
C	-7.256012	2.600905	6.603693
H	-6.047292	3.024826	4.220987
C	-8.644071	5.939307	-1.400244
H	-10.415646	6.025971	-0.156690

C	-7.322872	5.429633	-1.498673
C	-6.787458	4.694678	-0.404525
C	-6.584014	5.674492	-2.682019
H	-5.788911	4.278420	-0.490453
C	-8.011270	2.378220	7.729899
H	-6.201166	2.336309	6.587088
H	-7.558279	1.935497	8.611068
C	-9.387323	2.696557	7.731530
C	-9.977440	3.231347	6.611397
H	-9.980584	2.505764	8.620610
H	-11.037521	3.472694	6.607459
C	-7.122037	6.404992	-3.715191
H	-5.572400	5.287050	-2.768824
H	-6.531295	6.600267	-4.605332
C	-8.439579	6.906563	-3.615132
C	-9.183095	6.674396	-2.483626
H	-8.862277	7.476021	-4.437221
H	-10.200036	7.049884	-2.403272
C	-5.549396	0.310676	1.642569
C	-6.475153	1.346283	1.704400
H	-5.879665	-0.722570	1.675825
C	-6.041718	2.707809	1.672236
C	-4.685549	3.011267	1.586349
H	-4.350158	4.042557	1.604506
C	-1.930277	-2.304499	1.570278
C	-1.963658	-3.767678	1.564064
N	-3.732438	-7.014457	1.837075
C	-0.630413	-4.196701	1.589538
N	-1.054370	-7.855320	1.858820
C	0.201784	-2.994672	1.582425
N	-3.011094	-1.531417	1.579821
N	-0.623187	-1.907832	1.577230
C	-0.528050	-8.313663	3.086447
C	-1.433585	-8.747724	0.832706
C	-1.384734	-10.155404	1.084693
C	-0.505671	-9.727680	3.323311
N	-0.966108	-10.580202	2.331285
C	-1.761565	-11.044033	0.107395

C	-0.067693	-10.220006	4.525266
H	-0.756285	-11.561769	2.441618
C	-4.480163	-7.504964	0.741638
C	-3.740728	-7.693725	3.076344
C	-4.136632	-9.068869	3.101869
C	-4.960129	-8.853077	0.815837
N	-4.631666	-9.609114	1.931171
C	-3.992047	-9.802143	4.252770
C	-5.676323	-9.387841	-0.223424
H	-4.932981	-10.572380	1.945067
C	-2.219906	-10.605577	-1.162244
H	-1.717952	-12.110825	0.319070
C	-2.647908	-11.498335	-2.177274
C	-2.259034	-9.210502	-1.420325
C	-1.826828	-8.314269	-0.406607
H	-1.861156	-7.251284	-0.616590
C	-3.108064	-11.021876	-3.382838
H	-2.619298	-12.567957	-1.980447
H	-3.438479	-11.716835	-4.149561
C	-3.157980	-9.631793	-3.634970
C	-2.735398	-8.748693	-2.672020
H	-3.521628	-9.263314	-4.589022
H	-2.763643	-7.677953	-2.859274
C	0.392349	-9.364683	5.555540
H	-0.071230	-11.295228	4.689855
C	0.843563	-9.852208	6.806088
C	0.397401	-7.966273	5.318292
C	-0.079066	-7.472966	4.075189
H	-0.107676	-6.398064	3.937384
C	1.283020	-8.987854	7.780132
H	0.832098	-10.924871	6.983223
H	1.627059	-9.373381	8.735018
C	1.296342	-7.594766	7.541557
C	0.861865	-7.098150	6.336147
H	1.652261	-6.918957	8.313085
H	0.865700	-6.027129	6.146797
C	-3.463871	-9.222402	5.435858
H	-4.264201	-10.855403	4.252586

C	-3.141408	-7.838931	5.433425
C	-3.314590	-7.094849	4.233116
C	-2.600905	-7.256012	6.603693
H	-3.024826	-6.047292	4.220987
C	-5.939307	-8.644071	-1.400244
H	-6.025971	-10.415646	-0.156690
C	-5.429633	-7.322872	-1.498673
C	-4.694678	-6.787458	-0.404525
C	-5.674492	-6.584014	-2.682019
H	-4.278420	-5.788911	-0.490453
C	-2.378220	-8.011270	7.729899
H	-2.336309	-6.201166	6.587088
H	-1.935497	-7.558279	8.611068
C	-2.696557	-9.387323	7.731530
C	-3.231347	-9.977440	6.611397
H	-2.505764	-9.980584	8.620610
H	-3.472694	-11.037521	6.607459
C	-6.404992	-7.122037	-3.715191
H	-5.287050	-5.572400	-2.768824
H	-6.600267	-6.531295	-4.605332
C	-6.906563	-8.439579	-3.615132
C	-6.674396	-9.183095	-2.483626
H	-7.476021	-8.862277	-4.437221
H	-7.049884	-10.200036	-2.403272
C	-0.310676	-5.549396	1.642569
C	-1.346283	-6.475153	1.704400
H	0.722570	-5.879665	1.675825
C	-2.707809	-6.041718	1.672236
C	-3.011267	-4.685549	1.586349
H	-4.042557	-4.350158	1.604506
C	2.304499	-1.930277	1.570278
C	3.767678	-1.963658	1.564064
N	7.014457	-3.732438	1.837075
C	4.196701	-0.630413	1.589538
N	7.855320	-1.054370	1.858820
C	2.994672	0.201784	1.582425
N	1.531417	-3.011094	1.579821
N	1.907832	-0.623187	1.577230

C	8.313663	-0.528050	3.086447
C	8.747724	-1.433585	0.832706
C	10.155404	-1.384734	1.084693
C	9.727680	-0.505671	3.323311
N	10.580202	-0.966108	2.331285
C	11.044033	-1.761565	0.107395
C	10.220006	-0.067693	4.525266
H	11.561769	-0.756285	2.441618
C	7.504964	-4.480163	0.741638
C	7.693725	-3.740728	3.076344
C	9.068869	-4.136632	3.101869
C	8.853077	-4.960129	0.815837
N	9.609114	-4.631666	1.931171
C	9.802143	-3.992047	4.252770
C	9.387841	-5.676323	-0.223424
H	10.572380	-4.932981	1.945067
C	10.605577	-2.219906	-1.162244
H	12.110825	-1.717952	0.319070
C	11.498335	-2.647908	-2.177274
C	9.210502	-2.259034	-1.420325
C	8.314269	-1.826828	-0.406607
H	7.251284	-1.861156	-0.616590
C	11.021876	-3.108064	-3.382838
H	12.567957	-2.619298	-1.980447
H	11.716835	-3.438479	-4.149561
C	9.631793	-3.157980	-3.634970
C	8.748693	-2.735398	-2.672020
H	9.263314	-3.521628	-4.589022
H	7.677953	-2.763643	-2.859274
C	9.364683	0.392349	5.555540
H	11.295228	-0.071230	4.689855
C	9.852208	0.843563	6.806088
C	7.966273	0.397401	5.318292
C	7.472966	-0.079066	4.075189
H	6.398064	-0.107676	3.937384
C	8.987854	1.283020	7.780132
H	10.924871	0.832098	6.983223
H	9.373381	1.627059	8.735018

C	7.594766	1.296342	7.541557
C	7.098150	0.861865	6.336147
H	6.918957	1.652261	8.313085
H	6.027129	0.865700	6.146797
C	9.222402	-3.463871	5.435858
H	10.855403	-4.264201	4.252586
C	7.838931	-3.141408	5.433425
C	7.094849	-3.314590	4.233116
C	7.256012	-2.600905	6.603693
H	6.047292	-3.024826	4.220987
C	8.644071	-5.939307	-1.400244
H	10.415646	-6.025971	-0.156690
C	7.322872	-5.429633	-1.498673
C	6.787458	-4.694678	-0.404525
C	6.584014	-5.674492	-2.682019
H	5.788911	-4.278420	-0.490453
C	8.011270	-2.378220	7.729899
H	6.201166	-2.336309	6.587088
H	7.558279	-1.935497	8.611068
C	9.387323	-2.696557	7.731530
C	9.977440	-3.231347	6.611397
H	9.980584	-2.505764	8.620610
H	11.037521	-3.472694	6.607459
C	7.122037	-6.404992	-3.715191
H	5.572400	-5.287050	-2.768824
H	6.531295	-6.600267	-4.605332
C	8.439579	-6.906563	-3.615132
C	9.183095	-6.674396	-2.483626
H	8.862277	-7.476021	-4.437221
H	10.200036	-7.049884	-2.403272
C	5.549396	-0.310676	1.642569
C	6.475153	-1.346283	1.704400
H	5.879665	0.722570	1.675825
C	6.041718	-2.707809	1.672236
C	4.685549	-3.011267	1.586349
H	4.350158	-4.042557	1.604506
Zn	0.000000	0.000000	1.674967
C	2.994672	-0.201784	-1.582425

C	4.196701	0.630413	-1.589538
N	7.855320	1.054370	-1.858820
C	3.767678	1.963658	-1.564064
N	7.014457	3.732438	-1.837075
C	2.304499	1.930277	-1.570278
N	3.011094	-1.531417	-1.579821
N	1.907832	0.623187	-1.577230
C	7.504964	4.480163	-0.741638
C	7.693725	3.740728	-3.076344
C	9.068869	4.136632	-3.101869
C	8.853077	4.960129	-0.815837
N	9.609114	4.631666	-1.931171
C	9.802143	3.992047	-4.252770
C	9.387841	5.676323	0.223424
H	10.572380	4.932981	-1.945067
C	8.313663	0.528050	-3.086447
C	8.747724	1.433585	-0.832706
C	10.155404	1.384734	-1.084693
C	9.727680	0.505671	-3.323311
N	10.580202	0.966108	-2.331285
C	11.044033	1.761565	-0.107395
C	10.220006	0.067693	-4.525266
H	11.561769	0.756285	-2.441618
C	9.222402	3.463871	-5.435858
H	10.855403	4.264201	-4.252586
C	9.977440	3.231347	-6.611397
C	7.838931	3.141408	-5.433425
C	7.094849	3.314590	-4.233116
H	6.047292	3.024826	-4.220987
C	9.387323	2.696557	-7.731530
H	11.037521	3.472694	-6.607459
H	9.980584	2.505764	-8.620610
C	8.011270	2.378220	-7.729899
C	7.256012	2.600905	-6.603693
H	7.558279	1.935497	-8.611068
H	6.201166	2.336309	-6.587088
C	8.644071	5.939307	1.400244
H	10.415646	6.025971	0.156690

C	9.183095	6.674396	2.483626
C	7.322872	5.429633	1.498673
C	6.787458	4.694678	0.404525
H	5.788911	4.278420	0.490453
C	8.439579	6.906563	3.615132
H	10.200036	7.049884	2.403272
H	8.862277	7.476021	4.437221
C	7.122037	6.404992	3.715191
C	6.584014	5.674492	2.682019
H	6.531295	6.600267	4.605332
H	5.572400	5.287050	2.768824
C	10.605577	2.219906	1.162244
H	12.110825	1.717952	-0.319070
C	9.210502	2.259034	1.420325
C	8.314269	1.826828	0.406607
C	8.748693	2.735398	2.672020
H	7.251284	1.861156	0.616590
C	9.364683	-0.392349	-5.555540
H	11.295228	0.071230	-4.689855
C	7.966273	-0.397401	-5.318292
C	7.472966	0.079066	-4.075189
C	7.098150	-0.861865	-6.336147
H	6.398064	0.107676	-3.937384
C	9.631793	3.157980	3.634970
H	7.677953	2.763643	2.859274
H	9.263314	3.521628	4.589022
C	11.021876	3.108064	3.382838
C	11.498335	2.647908	2.177274
H	11.716835	3.438479	4.149561
H	12.567957	2.619298	1.980447
C	7.594766	-1.296342	-7.541557
H	6.027129	-0.865700	-6.146797
H	6.918957	-1.652261	-8.313085
C	8.987854	-1.283020	-7.780132
C	9.852208	-0.843563	-6.806088
H	9.373381	-1.627059	-8.735018
H	10.924871	-0.832098	-6.983223
C	4.685549	3.011267	-1.586349

C	6.041718	2.707809	-1.672236
H	4.350158	4.042557	-1.604506
C	6.475153	1.346283	-1.704400
C	5.549396	0.310676	-1.642569
H	5.879665	-0.722570	-1.675825
C	0.201784	2.994672	-1.582425
C	-0.630413	4.196701	-1.589538
N	-1.054370	7.855320	-1.858820
C	-1.963658	3.767678	-1.564064
N	-3.732438	7.014457	-1.837075
C	-1.930277	2.304499	-1.570278
N	1.531417	3.011094	-1.579821
N	-0.623187	1.907832	-1.577230
C	-4.480163	7.504964	-0.741638
C	-3.740728	7.693725	-3.076344
C	-4.136632	9.068869	-3.101869
C	-4.960129	8.853077	-0.815837
N	-4.631666	9.609114	-1.931171
C	-3.992047	9.802143	-4.252770
C	-5.676323	9.387841	0.223424
H	-4.932981	10.572380	-1.945067
C	-0.528050	8.313663	-3.086447
C	-1.433585	8.747724	-0.832706
C	-1.384734	10.155404	-1.084693
C	-0.505671	9.727680	-3.323311
N	-0.966108	10.580202	-2.331285
C	-1.761565	11.044033	-0.107395
C	-0.067693	10.220006	-4.525266
H	-0.756285	11.561769	-2.441618
C	-3.463871	9.222402	-5.435858
H	-4.264201	10.855403	-4.252586
C	-3.231347	9.977440	-6.611397
C	-3.141408	7.838931	-5.433425
C	-3.314590	7.094849	-4.233116
H	-3.024826	6.047292	-4.220987
C	-2.696557	9.387323	-7.731530
H	-3.472694	11.037521	-6.607459
H	-2.505764	9.980584	-8.620610

C	-2.378220	8.011270	-7.729899
C	-2.600905	7.256012	-6.603693
H	-1.935497	7.558279	-8.611068
H	-2.336309	6.201166	-6.587088
C	-5.939307	8.644071	1.400244
H	-6.025971	10.415646	0.156690
C	-6.674396	9.183095	2.483626
C	-5.429633	7.322872	1.498673
C	-4.694678	6.787458	0.404525
H	-4.278420	5.788911	0.490453
C	-6.906563	8.439579	3.615132
H	-7.049884	10.200036	2.403272
H	-7.476021	8.862277	4.437221
C	-6.404992	7.122037	3.715191
C	-5.674492	6.584014	2.682019
H	-6.600267	6.531295	4.605332
H	-5.287050	5.572400	2.768824
C	-2.219906	10.605577	1.162244
H	-1.717952	12.110825	-0.319070
C	-2.259034	9.210502	1.420325
C	-1.826828	8.314269	0.406607
C	-2.735398	8.748693	2.672020
H	-1.861156	7.251284	0.616590
C	0.392349	9.364683	-5.555540
H	-0.071230	11.295228	-4.689855
C	0.397401	7.966273	-5.318292
C	-0.079066	7.472966	-4.075189
C	0.861865	7.098150	-6.336147
H	-0.107676	6.398064	-3.937384
C	-3.157980	9.631793	3.634970
H	-2.763643	7.677953	2.859274
H	-3.521628	9.263314	4.589022
C	-3.108064	11.021876	3.382838
C	-2.647908	11.498335	2.177274
H	-3.438479	11.716835	4.149561
H	-2.619298	12.567957	1.980447
C	1.296342	7.594766	-7.541557
H	0.865700	6.027129	-6.146797

H	1.652261	6.918957	-8.313085
C	1.283020	8.987854	-7.780132
C	0.843563	9.852208	-6.806088
H	1.627059	9.373381	-8.735018
H	0.832098	10.924871	-6.983223
C	-3.011267	4.685549	-1.586349
C	-2.707809	6.041718	-1.672236
H	-4.042557	4.350158	-1.604506
C	-1.346283	6.475153	-1.704400
C	-0.310676	5.549396	-1.642569
H	0.722570	5.879665	-1.675825
C	-2.994672	0.201784	-1.582425
C	-4.196701	-0.630413	-1.589538
N	-7.855320	-1.054370	-1.858820
C	-3.767678	-1.963658	-1.564064
N	-7.014457	-3.732438	-1.837075
C	-2.304499	-1.930277	-1.570278
N	-3.011094	1.531417	-1.579821
N	-1.907832	-0.623187	-1.577230
C	-7.504964	-4.480163	-0.741638
C	-7.693725	-3.740728	-3.076344
C	-9.068869	-4.136632	-3.101869
C	-8.853077	-4.960129	-0.815837
N	-9.609114	-4.631666	-1.931171
C	-9.802143	-3.992047	-4.252770
C	-9.387841	-5.676323	0.223424
H	-10.572380	-4.932981	-1.945067
C	-8.313663	-0.528050	-3.086447
C	-8.747724	-1.433585	-0.832706
C	-10.155404	-1.384734	-1.084693
C	-9.727680	-0.505671	-3.323311
N	-10.580202	-0.966108	-2.331285
C	-11.044033	-1.761565	-0.107395
C	-10.220006	-0.067693	-4.525266
H	-11.561769	-0.756285	-2.441618
C	-9.222402	-3.463871	-5.435858
H	-10.855403	-4.264201	-4.252586
C	-9.977440	-3.231347	-6.611397

C	-7.838931	-3.141408	-5.433425
C	-7.094849	-3.314590	-4.233116
H	-6.047292	-3.024826	-4.220987
C	-9.387323	-2.696557	-7.731530
H	-11.037521	-3.472694	-6.607459
H	-9.980584	-2.505764	-8.620610
C	-8.011270	-2.378220	-7.729899
C	-7.256012	-2.600905	-6.603693
H	-7.558279	-1.935497	-8.611068
H	-6.201166	-2.336309	-6.587088
C	-8.644071	-5.939307	1.400244
H	-10.415646	-6.025971	0.156690
C	-9.183095	-6.674396	2.483626
C	-7.322872	-5.429633	1.498673
C	-6.787458	-4.694678	0.404525
H	-5.788911	-4.278420	0.490453
C	-8.439579	-6.906563	3.615132
H	-10.200036	-7.049884	2.403272
H	-8.862277	-7.476021	4.437221
C	-7.122037	-6.404992	3.715191
C	-6.584014	-5.674492	2.682019
H	-6.531295	-6.600267	4.605332
H	-5.572400	-5.287050	2.768824
C	-10.605577	-2.219906	1.162244
H	-12.110825	-1.717952	-0.319070
C	-9.210502	-2.259034	1.420325
C	-8.314269	-1.826828	0.406607
C	-8.748693	-2.735398	2.672020
H	-7.251284	-1.861156	0.616590
C	-9.364683	0.392349	-5.555540
H	-11.295228	-0.071230	-4.689855
C	-7.966273	0.397401	-5.318292
C	-7.472966	-0.079066	-4.075189
C	-7.098150	0.861865	-6.336147
H	-6.398064	-0.107676	-3.937384
C	-9.631793	-3.157980	3.634970
H	-7.677953	-2.763643	2.859274
H	-9.263314	-3.521628	4.589022

C	-11.021876	-3.108064	3.382838
C	-11.498335	-2.647908	2.177274
H	-11.716835	-3.438479	4.149561
H	-12.567957	-2.619298	1.980447
C	-7.594766	1.296342	-7.541557
H	-6.027129	0.865700	-6.146797
H	-6.918957	1.652261	-8.313085
C	-8.987854	1.283020	-7.780132
C	-9.852208	0.843563	-6.806088
H	-9.373381	1.627059	-8.735018
H	-10.924871	0.832098	-6.983223
C	-4.685549	-3.011267	-1.586349
C	-6.041718	-2.707809	-1.672236
H	-4.350158	-4.042557	-1.604506
C	-6.475153	-1.346283	-1.704400
C	-5.549396	-0.310676	-1.642569
H	-5.879665	0.722570	-1.675825
C	-0.201784	-2.994672	-1.582425
C	0.630413	-4.196701	-1.589538
N	1.054370	-7.855320	-1.858820
C	1.963658	-3.767678	-1.564064
N	3.732438	-7.014457	-1.837075
C	1.930277	-2.304499	-1.570278
N	-1.531417	-3.011094	-1.579821
N	0.623187	-1.907832	-1.577230
C	4.480163	-7.504964	-0.741638
C	3.740728	-7.693725	-3.076344
C	4.136632	-9.068869	-3.101869
C	4.960129	-8.853077	-0.815837
N	4.631666	-9.609114	-1.931171
C	3.992047	-9.802143	-4.252770
C	5.676323	-9.387841	0.223424
H	4.932981	-10.572380	-1.945067
C	0.528050	-8.313663	-3.086447
C	1.433585	-8.747724	-0.832706
C	1.384734	-10.155404	-1.084693
C	0.505671	-9.727680	-3.323311
N	0.966108	-10.580202	-2.331285

C	1.761565	-11.044033	-0.107395
C	0.067693	-10.220006	-4.525266
H	0.756285	-11.561769	-2.441618
C	3.463871	-9.222402	-5.435858
H	4.264201	-10.855403	-4.252586
C	3.231347	-9.977440	-6.611397
C	3.141408	-7.838931	-5.433425
C	3.314590	-7.094849	-4.233116
H	3.024826	-6.047292	-4.220987
C	2.696557	-9.387323	-7.731530
H	3.472694	-11.037521	-6.607459
H	2.505764	-9.980584	-8.620610
C	2.378220	-8.011270	-7.729899
C	2.600905	-7.256012	-6.603693
H	1.935497	-7.558279	-8.611068
H	2.336309	-6.201166	-6.587088
C	5.939307	-8.644071	1.400244
H	6.025971	-10.415646	0.156690
C	6.674396	-9.183095	2.483626
C	5.429633	-7.322872	1.498673
C	4.694678	-6.787458	0.404525
H	4.278420	-5.788911	0.490453
C	6.906563	-8.439579	3.615132
H	7.049884	-10.200036	2.403272
H	7.476021	-8.862277	4.437221
C	6.404992	-7.122037	3.715191
C	5.674492	-6.584014	2.682019
H	6.600267	-6.531295	4.605332
H	5.287050	-5.572400	2.768824
C	2.219906	-10.605577	1.162244
H	1.717952	-12.110825	-0.319070
C	2.259034	-9.210502	1.420325
C	1.826828	-8.314269	0.406607
C	2.735398	-8.748693	2.672020
H	1.861156	-7.251284	0.616590
C	-0.392349	-9.364683	-5.555540
H	0.071230	-11.295228	-4.689855
C	-0.397401	-7.966273	-5.318292

C	0.079066	-7.472966	-4.075189
C	-0.861865	-7.098150	-6.336147
H	0.107676	-6.398064	-3.937384
C	3.157980	-9.631793	3.634970
H	2.763643	-7.677953	2.859274
H	3.521628	-9.263314	4.589022
C	3.108064	-11.021876	3.382838
C	2.647908	-11.498335	2.177274
H	3.438479	-11.716835	4.149561
H	2.619298	-12.567957	1.980447
C	-1.296342	-7.594766	-7.541557
H	-0.865700	-6.027129	-6.146797
H	-1.652261	-6.918957	-8.313085
C	-1.283020	-8.987854	-7.780132
C	-0.843563	-9.852208	-6.806088
H	-1.627059	-9.373381	-8.735018
H	-0.832098	-10.924871	-6.983223
C	3.011267	-4.685549	-1.586349
C	2.707809	-6.041718	-1.672236
H	4.042557	-4.350158	-1.604506
C	1.346283	-6.475153	-1.704400
C	0.310676	-5.549396	-1.642569
H	-0.722570	-5.879665	-1.675825
Zn	0.000000	0.000000	-1.674967
